# Database-Driven Identification of Structurally Similar Protein-Protein Interfaces

**DOI:** 10.1021/acs.jcim.3c01462

**Published:** 2024-03-12

**Authors:** Joel Graef, Christiane Ehrt, Thorben Reim, Matthias Rarey

**Affiliations:** Universität Hamburg, ZBH—Center for Bioinformatics , Albert-Einstein-Ring 8-10, 22761 Hamburg, Germany

## Abstract

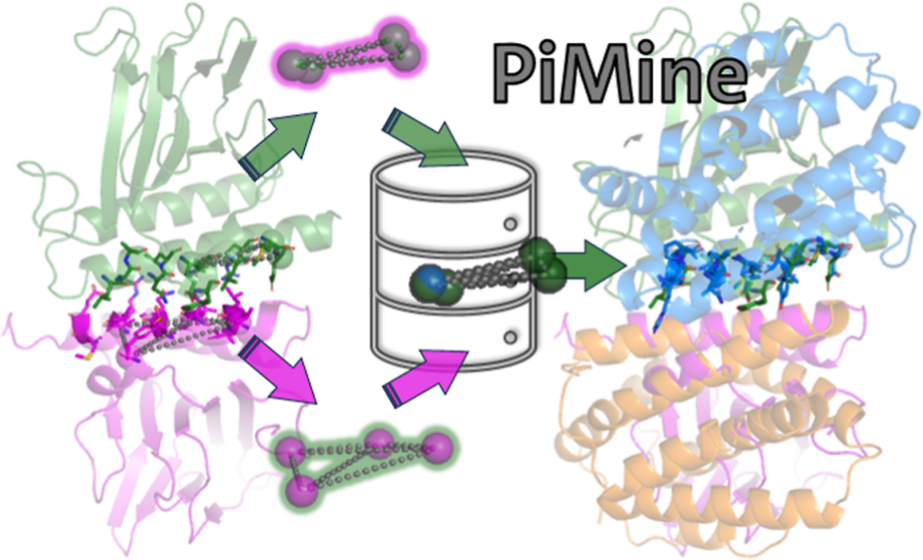

Analyzing the similarity of protein interfaces in protein-protein interactions gives new insights into protein function and assists in discovering new drugs. Usually, tools that assess the similarity focus on the interactions between two protein interfaces, while sometimes we only have one predicted interface. Herein, we present PiMine, a database-driven protein interface similarity search. It compares interface residues of one or two interacting chains by calculating and searching tetrahedral geometric patterns of α-carbon atoms and calculating physicochemical and shape-based similarity. On a dedicated, tailor-made dataset, we show that PiMine outperforms commonly used comparison tools in terms of early enrichment when considering interfaces of sequentially and structurally unrelated proteins. In an application example, we demonstrate its usability for protein interaction partner prediction by comparing predicted interfaces to known protein-protein interfaces.

## Introduction

Protein-protein interactions (PPIs) are integral for many cellular processes and are associated with various diseases.^1^ Targeting PPIs represents a major challenge with high potential for drug discovery.^2^ An interesting illustration of how known protein-protein complexes can be used for the rational design of drugs is Venetoclax, which was approved for the therapy of chronic lymphocytic leukemia in 2016.^3^ Its predecessor molecules, ABT-737^4^ and ABT-263 (Navitoclax),^5^ were designed based on a known complex structure of its antiapoptotic target BCL-X_L_ and a peptide derived from the pro-apoptotic protein Bak. The investigation of the structures of these inhibitors in complex with BCL-2 and a re-engineering strategy led to the development of Venetoclax,^6^ impressively illustrating the impact of our knowledge of PPIs for drug design. However, in contrast to the exploitation of the comparatively small protein binding pockets, there are still few computational methods for targeting protein-protein interfaces, which are called interfaces for simplicity. This lack of in silico tools can be attributed to multiple reasons, such as interfaces being large with 1500–3000 Å^2^, very hydrophobic, and flat without deep cavities. Therefore, designing small-molecule binders is difficult.^7^ Although permanent and transient complexes differ in these properties,^8^ both types of PPIs could be successfully addressed by small molecule binders in the past.^9^ Therefore, we do not distinguish between these PPI types, which are both challenging to address. Nevertheless, the analysis of interfaces and the interactions can lead to insights into, e.g., the affinity of interactions, the biological function, or potential side effects of PPI-based drugs due to interface similarities. Although we have abundant information on biologically relevant PPIs based on experimental data,^10^ their protein-protein complex structure is often unknown. Moreover, proteins of unknown function might harbor interfaces for PPI, whose knowledge might help unravel their biological impact.^11^ Also, known small molecule inhibitors of similar PPIs can be explored by researchers to study their interfaces.^12^ Comparing interfaces and looking for similarities is one approach to broadening our structural knowledge of interfaces by analyzing protein sequences and their structures. While numerous sequences are known, the generated alignments of sequence-dependent methods always obey the sequence order. As many functionally similar binding interfaces are sequence-independent,^13^ the latter methods fail to detect their similarities. Also, chains with similar interfaces might be structurally close but sequentially remote. In these cases, structure-dependent methods are the solution of choice, provided a protein structure model is available. The prediction of interfaces and a comparison to already known biologically relevant interfaces can help in understanding the structural details of PPIs. Therefore, interface comparison methods should enable users to screen databases based on predicted interfaces for single-chain protein structures.^14^ This possibility is often not implemented in current comparison approaches. Furthermore, programs for similarity assessment differ in the interface region definition, the algorithm, the applied scoring functions, and the datasets on which the methods are parametrized and tested.

Tools for structure-based calculation of protein-protein interface similarities are usually developed as standalone programs, performing pairwise comparisons. In contrast, structurally comparing one interface to, e.g., the complete Protein Data Bank (PDB),^15^ is considerably more time-consuming than sequence-based approaches. To our knowledge, the tool I2I-SiteEngine^16,17^ was the only protein-protein interface similarity search still available online as a web server. However, the analysis was restricted to pairwise comparisons. Unfortunately, the standalone tool and web server are no longer accessible at the time of submission.

Table 1 gives an exemplary selection of protein-protein interface similarity calculation methods. iAlign^18^ shares a similar concept to the protein structure alignment method TM-align.^19^ It defines protein-protein interfaces by the heavy atom distances between protein residues. Thus, a residue is part of an interface if at least one of its heavy atoms is within a maximum distance of 4.5 Å from any heavy atom of the other protein chain. Nevertheless, the interface comparisons use complete protein sequences. Two scoring functions are available: the TM-score and the IS-score. Both estimate the similarity based on the Cα atoms of the aligned residue pairs. In addition to the pairwise distances, the IS-score includes a so-called contact overlap factor. It describes the conservation of interfacial contact patterns. The alignment algorithm calculates three initial alignments: gapless threading, secondary structure, and fragment assembly alignments. These alignments result in a scoring matrix for iterative refinements. The algorithm stops after 30 iterations or when the alignment converges. A drawback of this method is that the alignments are still-dependent on the quality of the sequence alignments, as it relies on gapless alignments in the initial steps.

**Table 1:** Exemplary Selection of Tools for Protein-Protein Interface Similarity Calculations

method	availability	citation count	algorithmic approach
I2I-SiteEngine^16^	not available	21 (92^a^)	interface definition: 4 Å; representation of interface as surface points that describe physicochemical properties and local surface curvature; triangles of surface points are hashed and used for matching
CMAPi^22^	not available	22	interface definition: 10 Å; interfaces as contact map matrices of residues; uses 2D dynamic programming to optimize alignment score using the Smith-Waterman^23^ algorithm
iAlign^18^	standalone	75	interface definition: 4.5 Å; three initial alignments are calculated using gapless threading, secondary structure, and fragment assembly; alignments are refined using dynamic programming
PCalign^24^	standalone	15	interface definition: 4.5 Å; Cα atoms are applied to geometric hashing where the Cα atoms are assigned with chemical types of the residue and an alignment of those point clouds is searched
PROSTA-inter^25^	standalone^b^	15	interface definition: 6 Å; selects Cα atoms and calculates alignments based on local and remote fragments; alignments are clustered and further refined for final results; also supports nucleic acids (C3′ atoms)
InterComp^26^	standalone	13	interface definition: 5 Å; represents interface residues as points in space using Cα atoms; uses simulated annealing and compares distance maps; sequence-dependent
PatchBag^27^	standalone	3	bag-of-words approach which represents the protein surface or interfaces as vectors of counts of geometrical types of surface patches; patches are defined for a residue by its Cα atom and 4 neighboring Cα atoms

a SiteEngine^17^ citation count.

b Web service not available anymore.

I2I-SiteEngine^16^ defines interfaces in the same way as iAlign, but with a maximum distance of 4 Å. The interfaces are described by surface points and are annotated by the physicochemical properties of the functional groups of the residues. Surface points are then grouped into surface patches. In addition, a shape function based on solid angles describes the average curvature of each surface patch.^16,20,21^ Then, the centers of the patches are combined as multiple triangles and retained if they have complementary physicochemical properties to a patch in the second protein interface, with which they build up the protein-protein interface. The triangles are hashed and can be searched for in other protein complexes. Two scoring functions that use the physicochemical properties and the solid angles assess the interface similarity. While the initial score is applied to a low-resolution representation to reduce the search space, the second score uses a higher resolution level. The match list is then enumerated by calculating the maximum weight match using a bipartite graph. In this process, two so-called 1:1 correspondence scores are calculated. Finally, all scores are summed to get the total similarity score. The alignments of I2I-SiteEngine are fully sequence-independent, but the method has a much longer runtime than iAlign.^18^

In this study, we present PiMine, a tool for the alignment and similarity assessment of protein-protein interfaces based on structural features. Its algorithm assumes that if one protein chain or interface of a PPI is similar to one of another PPI, both interfaces are similar. In contrast to other interface-comparison tools, PiMine calculates the similarity for interfaces between two interacting protein chains as well as for one-sided interface regions, as defined by a user or predicted by a third-party program. The latter is a major benefit compared to other interface comparison methods. PiMine reports three similarity scores based on the shape, the physicochemical properties, and a combination thereof. In addition, the tool provides the aligned protein structures in the PDB format.

Here, we benchmark PiMine against the currently most cited ones for interface comparison based on both existing well-known datasets and newly developed datasets to evaluate screening performance, alignment quality, and runtime. Given the high early enrichment of PiMine for state-of-the-art datasets, we could confirm that our assumption that the similarity between two protein chains often also reflects the similarity of the interfaces of the two interacting chains holds for known similar interface pairs. Based on a novel, highly unbiased benchmark dataset, we demonstrate that PiMine performs superior to frequently cited tools in correctly detecting remote similarities between sequentially unrelated interfaces. Moreover, we show that a comparison based on the interfaces of single chains with PiMine is robust and leads to a convincing early enrichment of similar interface pairs. Finally, we illustrate how PiMine performs well for known application examples of interface comparison methods and how it can be applied to identify novel potential interaction partners of protein chains.

## Methods

### Interface Modeling

PiMine, like many other methods, determines the interface regions of PPIs by the proximity of protein chains. Protein heavy atoms within 4.5 Å of any heavy atom of the interacting chain constitute the interface.

### Alignment Algorithm

We developed the new algorithmic concept of TetraScan for binding site^28^ and protein-protein interface comparisons. Here, we describe the method in the context of interface similarity. TetraScan creates database queries for protein site atoms in the form of tetrahedrons. It uses a database based on the GeoMine technology^29^ comprising all interface atoms of protein–ligand or protein-protein complexes to compare. During database creation, reasonable protonation states^30^ are determined, hydrogen atom positions are predicted, and the interface atoms are stored along with their spatial coordinates and physicochemical properties.

**Figure 1:**
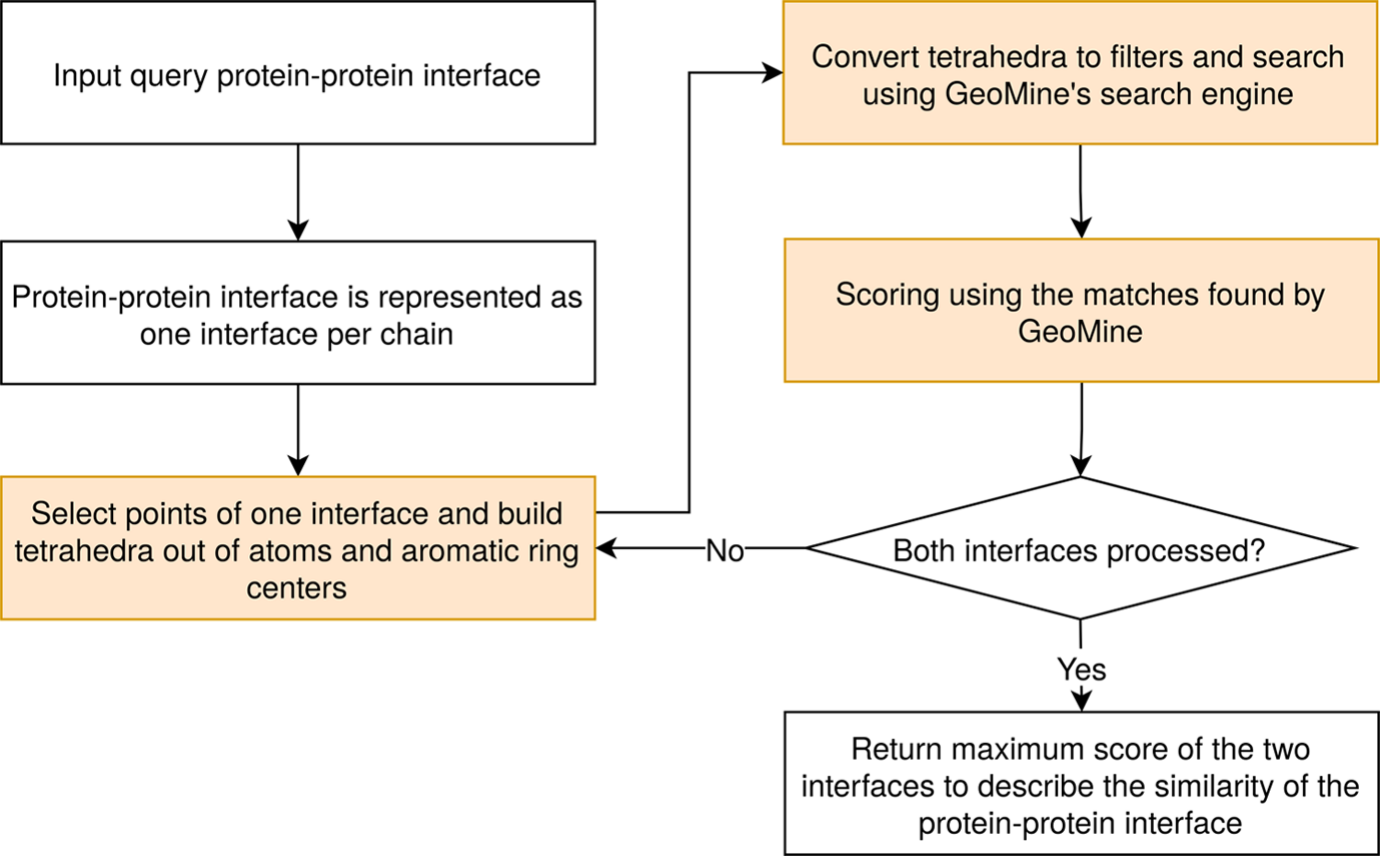
Overview of the PiMine algorithm steps illustrated as boxes. The TetraScan algorithm is highlighted in orange.

Figure 1 provides an overview of the search algorithm. It starts using a PiMine database created for complex structures in the PDB or mmCIF file format and a query protein-protein interface whose similarity to other protein-protein interfaces should be assessed. This query protein-protein interface consists of a subset of protein atoms of two nearby chains or predefined interface atoms of a single chain. The interface specification options are described in more detail in the Supporting Information (see Paragraph S1).

The interface atoms can optionally be reduced to only the Cα atoms of all residues (default) or to so-called “restricted” points, which are all heteroatoms (oxygen, nitrogen, and sulfur), all aromatic ring centers (His, Phe, Trp, and Tyr), and all hydrophobic side chain carbon atoms of Ala, Ile, Leu, Lys, Met, Pro, and Val. When using the restricted points or all points, the interface atoms are reduced to solvent-exposed atoms of the unbound structure. Aromatic ring centers are used irrespective of the solvent exposure of the corresponding ring atoms. Then, tetrahedra are generated for all interface atoms of a single chain. The distance between two atoms or corners of a tetrahedron must be in a specified range, optionally defined by the user (default: *minDist* = 1 Å, *maxDist* = 14 Å). The set of generated tetrahedra is sorted in descending order according to the sum of their edge lengths. From this set, the tetrahedrons are selected. The selection ensures that each interface atom is part of at least one tetrahedron. If all interface atoms are represented and more tetrahedrons than a defined value (default: *noffilters* = 30) are calculated, only the *noffilters* largest tetrahedrons are selected. The resulting tetrahedra are converted to filters for querying the database with PiMine. All edges are annotated by the distances between the atoms or aromatic centers and an adjustable tolerance (default: *distTolerance* = 1.0 Å). For the atoms, different properties such as residue name, residue class, backbone/side chain, solvent exposure, aromatic center, chemical element, and atom interaction type are annotated if available. The properties used in the search depend on the selected points and the chosen hierarchy level. Four hierarchy levels are available: (1) atom interaction type only, (2) chemical element and atom interaction type, (3) residue type, element, and backbone/side chain, and (4) residue name, chemical element, and backbone/side chain. By default, PiMine uses the second hierarchy level and handles Cα atoms as a unique atom type differentiated from other carbons (adjustable using the parameter *filterHierarchyLevel*).

The search returns a series of atom mappings for the query tetrahedrons. One hit represents a tetrahedron pattern from the query interface also occurring in the target interface, where all hierarchy-specific properties of the atoms and defined distance criteria match. The respective tetrahedron filter can match several times in the same target interface, matching even the same atoms. Symmetrical atom mappings are not filtered because they lead to different alignments and therefore different scores. For each hit, a superposition of the query and the match can be calculated with the Kabsch–Umeyama algorithm.^31−33^ We assess whether a hit provides a good superposition and thus has a high degree of similarity in two steps. First, a prefiltering step checks the shape score based on the Cα atoms and a radius of 6 Å. This radius definition represents the average residue diameter of about 10.6 Å^34^ and a small tolerance of 1.4 Å, leading to 12 Å. Second, the best *x* hits per matching target interface are selected, where *x* is defined as the square root of the number of total hits in the respective target interface. After this prefiltering step, the similarity scores for all remaining hits are calculated. These atom-wise scores are considering all neighboring atoms in a predefined radius (default: *scoringRadius* = 1.5 Å). For each target interface, the highest-scoring alignment is selected at the end.

Since the similarity determination may depend on the particular protein chain under consideration, the query interface should be chosen appropriately. A PPI is defined by one interface per protein chain. Thus, there are four possible chain pairs for the similarity calculation. For the query interface chains A and B and the target interface chains A′ and B′, four chain-based alignments are possible: (1) A versus A′, (2) A versus B′, (3) B versus A′, and (4) B versus B′. Therefore, we calculate all four alignments and select the highest-scoring alignment for transforming the target match to the query structure. Note that, by default, we report the maximum of the four calculated interface similarity scores. Optionally, PiMine can also consider the similarities between both interfaces if the parameter *twoSidedScoring* is enabled.

Finally, the matches are returned with their corresponding scores and the transformed target interfaces in the PDB format for ranking, visualizing, and investigating the detected similarity if required.

### Similarity Measure

There are three similarity scores in PiMine: shape-based (shape score), pharmacophore-based (pharma score), and their equally weighted sum called the SP-score. The scores are calculated for all solvent-exposed points using a linear search or the nanoflann implementation of the k-dimensional tree,^35^ depending on how large the interface is, to reduce runtime. A linear search will be performed if there are fewer than 100 solvent-exposed interface points. For each query interface point, the closest point of the matching target interface is searched within a defined scoring radius. If at least one point is found, the counter for the shape score is incremented by one, and the pharma score is incremented based on pharmacophore properties using a knowledge-driven scoring matrix (Table 2). After all query interface points have been processed, both scores are normalized by the number of surface points of the larger single-chain interface, providing the shape and pharmacophore-based score for each hit. At the end of this process, the best alignments are selected based on the highest SP-score of all hits for the target interface.

**Table 2:** Scoring Schema for Two Atoms Regarding Their Pharmacophore-Based Similarity^a^

	Acc/Don	Acc	Don	Aro	HyPhob	Cα	Pos/Don	Neg/Acc
Acc/Don	1	0.6	0.6	0	0	0	0.6	0.6
Acc		1	0	0	0	0	0	0.8
Don			1	0	0	0	0.8	0
Aro				1	0.8	0	0	0
HyPhob					1	0	0	0
Cα						1	0	0
Pos/Don							1	0
Neg/Acc								1

a Acc/Don: hydrogen bond acceptor and donor, Acc: hydrogen bond acceptor, Don: hydrogen bond donor, Aro: aromatic ring atom, HyPhob: hydrophobic atom, Cα: α-carbon atom, Pos/Don: hydrogen bond donor and positively charged, and Neg/Acc: hydrogen bond acceptor and negatively charged.

### Datasets

We used five datasets in our experiments (Table 3).

**Table 3:** Overview of the Composition and Purpose of the Five Datasets Used in This Publication^a^

name	biological interfaces	similarity criterion	#Act	#Inact	date	purpose
*ParamOptSet*	manually curated^40^	similarity to native protein-protein complex;^36^ actives: high- and medium-quality complexes; inactives: acceptable and incorrect complexes	129	5549	2015	parameter optimization
*Dimer597*(18)	interfacial energy according to Lu et al.^41^ below −12^42^	same SCOP assignment of at least one structural similarity according to TM-align^18^; minimum contact overlap ratio of 0.3	373	176,875	2010	enrichment assessment (sequentially and structurally related chains)
*Keskin*(39)	≥10 interface residues	geometric similarity of the position of Cα atoms; percent residue identity in the match; size similarity of the interfaces	4876	176,627	2004	enrichment assessment (structurally related interfaces)
*PiMineSet*	EPPIC^43^ predictions for ASU	sequential and structural similarity between two chains of both interfaces; sequential and structural dissimilarity between the other two chains of both interfaces; residue overlap of 0.6 and 0.8 of the aligned interface chains	77	2718	2022	enrichment and alignment assessment
*RunTimeSet*	EPPIC^43^ predictions for ASU	n/a	n/a	n/a	2022	runtime analyses and applications

a ASU, asymmetric unit; #Act, number of similar interfaces; #Inact, number of dissimilar interfaces.

The first dataset, the *ParamOptSet*, encompasses complex structures for the scoring quality assessment in protein-protein docking studies^36^ to classify correctly and incorrectly predicted protein-protein complex structures. We downloaded the complete set of predicted complex structures^37^ and preprocessed the datasets as follows: the PDB structures of the native complexes were downloaded and used as a query. Matches with all predicted complexes of high- and medium-quality ((0.3 ≤ *f*_nat_ < 0.5) and (LRMSD ≤ 5.0 Å or IRMSD ≤ 2 Å) or (*f*_nat_ ≥ 0.5) and (LRMSD > 1.0 Å and IRMSD > 1.0 Å)) were handled as similar interfaces (actives). Their scores should be higher than those of matches with predicted complexes of acceptable quality or incorrectly predicted complexes (inactives). Here, LRMSD (originally L_rms) denotes the root-mean-square deviation (rmsd) of the smaller protein compared with the native pose, IRMSD (originally I_rms) is the backbone rmsd of the interface residues, and *f*_nat_ is “defined as the number of native (correct) residue–residue contacts in the predicted complex divided by the number of contacts in the target complex”.^38^ To ensure a more realistic ratio of inactives to actives, we used only structures with a ratio of at least 20 (inactives/actives). Furthermore, we excluded complexes with interfaces between more than two chains. The final dataset contains predicted structures for 18 native protein-protein complexes (18 groups, 5678 structures) for parameter optimization.

Details regarding the second (*Dimer597*)^18^ and third (*Keskin*)^39^ sets, two published datasets from the literature, can be found in Paragraph S2 in the Supporting Information. Pairwise comparisons of the chains constituting the datasets’ interfaces were performed with TM-align.^18^

To test whether PiMine detects similarities between single-chain interfaces of sequentially and structurally dissimilar chains, we designed another dataset called *PiMineSet*. We downloaded all structures from the PDB as of March 15th, 2022. Next, we applied the standalone version of EPPIC^43^ to find biological interfaces in all asymmetric units of these structures, as downloaded from https://github.com/eppic-team/eppic on September 9th, 2021. We retained only PDB structures with a resolution of at most 2 Å and a free *R*-factor of at most 0.25 from this set of biological interfaces and ignored structures without these annotations. The remaining structures were sequence-culled using Linclust^44^ in the slower but more sensitive “cluster” mode, with a minimum sequence identity of 25%. Apart from that, default settings were used. Next, we compared the sequence-culled protein chains against all protein chains of biological interfaces in our PDB subset using Foldseek^45^ with default settings and a TM-score threshold of 0. To find interfaces with two structurally related chains and two structurally unrelated chains as similar pairs of our new dataset, we checked each interface chain in the sequence-culled set of biological interfaces for its similarities to other biologically relevant interfaces. An interface pair was retained for further analyses if it fulfilled all the following conditions: (1) one chain of the query interface had a TM-score of at least 0.5 to another chain of the target interface, (2) this chain had a TM-score below 0.5 to the other chain of the target interface, and (3) the other chains of both interfaces had a TM-score below 0.5. These preselected pairs of interfaces were processed with UCSF Chimera.^46^ Both proteins were loaded, and the chains with a TM-score of at least 0.5 were aligned using MatchMaker.^47^ All residues of the similar query chain within a 4 Å environment of the corresponding partner chains were selected to determine the percentage of overlapping residues of the query chain in both interfaces relative to all interface residues of the interfaces of both complexes (residue overlap). We visually inspected all interfaces with an overlap (residue intersection) of at least 60% relative to the number of residues of one interface and 80% relative to the number of residues of the other one to extract interesting similar interface pairs. Also, a more detailed analysis with TM-align^19^ was performed to compare all interface chains. The TM-score for two chains of each pair had to be higher than 0.75, while the score for the other two chains of the interfaces had to be below 0.5.Interface pairs fulfilling the TM-score criteria applied for the Foldseek analysis with a relative overlap of at most 5% constitute the dissimilar interfaces (inactives) in this dataset. The TM-scores and relative residue overlaps of the finally chosen similar and dissimilar interface pairs can be found in the corresponding repository.^48^ Figure S1 in the Supporting Information shows exemplary similar and dissimilar interfaces in this dataset. An additional benefit of this dataset is the availability of the corresponding alignments. Therefore, we also used this set to benchmark the alignment performance of PiMine compared with other commonly used protein-protein complex alignment methods.

To analyze the runtime, we built a fifth dataset, named *RunTimeSet*, by applying the EPPIC software^43^ on all protein structures in the PDB on October 28th, 2022, to predict biological protein-protein interfaces in the asymmetric unit of the PDB entries. This dataset contains 169,944 interfaces in 59,928 structures, which is suitable for screening with known and predicted protein-protein interfaces, e.g., to identify potential protein interaction partners. For the run-time analyses, we compared the interface between chains A and B of the randomly selected PDB entry 3t4m against all dataset interfaces.

### Usage of External Tools

I2I-SiteEngine was downloaded (http://bioinfo3d.cs.tau.ac.il/cgi-bin/pdownload/progdownload.pl/?pname=I2ISiteEngine, last access: September 27th, 2022) and installed using the Perl script “install_I2ISiteEngine.pl”. I2I-SiteEngine was run with the default parameters. From all calculated scores (low-resolution score, overall surface score, 1:1 correspondence curvature and distance score, and total score), we assessed the performance using the highest total score. The executable “pdb_trans_all_atoms.Linux” for applying the transformation matrices of the alignments to the target structures did not work. Therefore, we applied the reported transformation matrix to the target PDB structures using the tool *pdbset* of the CCP4 Software Suite^49^ (version 7.1). For the screening dataset, 169,769 of 169,944 interfaces could be correctly prepared (99.9%). Subsequently, 169,714 interfaces were compared, while only 164,321 interfaces were aligned. All interfaces of the *RunTimeSet* were prepared beforehand, and we only measured the time for the comparisons. iAlign (version 1.1) was downloaded (https://sites.gatech.edu/cssb/ialign/) as a precompiled executable. The method was executed with the default parameters. However, the minimum number of residues for a protein chain and an interface was set to 3 to account for protein–peptide interfaces that are part of the numerous datasets analyzed herein. Also, we tested both available scoring functions in this work: the IS-score (default, iAlign-IS) and the TM-score (iAlign-TM). For the *RunTimeSet*, 6628 interfaces were not correctly detected due to lowercase chain identifiers that iAlign cannot process (3.9%). For the remaining set, 163,274 interfaces were successfully parsed. Besides the missing interfaces between chains with lower-case IDs, some interfaces, e.g., the interface between chains B and C of PDB entry 8eav (structures with unknown sequences, i.e., containing residues with three-letter code UNK), could not be found. Altogether, 163,254 alignments were obtained. For the run-time analyses, all PDB files were prepared beforehand, and the interfaces of the *RunTimeSet* were extracted. Only the runtime for the comparisons was measured.

### Parameter Optimization

The parameters of PiMine were optimized on the *ParamOptSet* introduced above. Based on this dataset, we evaluated the parameters maximum distance (*maxDist*), minimum distance (*minDist*), distance tolerance (*distTolerance*), and scoring radius (*scoringRadius*) (see section Alignment Algorithm). The maximum distance was varied between 11 and 14 Å in 0.5 Å steps. This range ensures that filters are generated for all interfaces in the *RunTimeSet*. To evaluate this, we varied the maximum distance and looked at the number of generated filters. For a maximum distance below 11 Å, no filters were generated for some interfaces. The minimum distance was increased from 1 to 3 Å in steps of 0.5 Å, while the distance tolerance values were tested from 0.5 to 1.5 Å in 0.5 Å steps. The scoring radius was varied between 1.25 and 2.0 Å in 0.25 Å steps. Besides that, we decided to use α-carbon atoms for filter generation as the default instead of also looking at other atoms to improve the runtime. Most comparison tools, e.g., InterComp^26^ and PatchBag,^27^ showed good performance although relying solely on α-carbon atoms. Also, we set the default number of generated filters to 30. It is used to select at least as many filters as are required to cover the complete modeled query protein interface. The second filter hierarchy level is used by default, which describes the selected atoms’ chemical elements and properties (e.g., hydrogen bond donor or hydrogen bond acceptor). Consequently, we investigated 7 × 5 × 3 × 4 = 420 parameter combinations.

The parameter combination results were first sorted according to their non-normalized enrichment factor (EF) values at 1, 2, 5, 10, and 20%, second to the area under the receiver operating characteristics (ROC) curve (AUC), and third to the runtime. Considering all of the results, the change in the minimum distance has no impact. On the other hand, the performance increases with increasing maximum distance, distance tolerance, and scoring radius. The results for the best eight parameter combination sets are listed in Table 4. All further parameter sets lead to values of 9.43 or lower for the EF at 1%.

**Table 4:** First Eight Results of the Optimization of the Maximum Distance (*maxDist*), the Minimum Distance (*minDist*), the Distance Tolerance (*distTolerance*), and the Scoring Radius (*scoringRadius*)^a^

entry	*minDist*	*maxDist*	*scoringRadius*	*distTolerance*	EF (1%, 2%, 5%, 10%, 20%)	AUC	time [s]
1	3	14	1.5	1.5	10.22, 6.62, 3.73, 2.64, 2.17	0.702	8825
2	2	14	1.5	1.5	10.22, 6.62, 3.73, 2.64, 2.17	0.701	5016
3	1	14	1.5	1.5	10.22, 6.62, 3.73, 2.64, 2.17	0.701	5589
4	1.5	14	1.5	1.5	10.22, 6.62, 3.73, 2.64, 2.17	0.701	5651
5	2.5	14	1.5	1.5	10.22, 6.62, 3.73, 2.64, 2.17	0.701	8860
6	3	14	1.5	1.0	10.22, 6.62, 3.73, 2.64, 2.13	0.686	7757
7	1	14	1.5	1.0	10.22, 6.62, 3.73, 2.64, 2.13	0.686	4761
8	2	14	1.5	1.0	10.22, 6.62, 3.73, 2.64, 2.13	0.686	4964

a Results are sorted by their enrichment factors (EFs), area under the receiver operating characteristics curve (AUC), and runtime.

The parameter sets of 1 to 5 lead to the same EFs and only show a slight AUC difference of 0.001. Entries 6 to 8 lead to a slight decrease of the EF at 20% (2.17 to 2.13) and a lower AUC. Results 7 and 8 had the shortest runtimes of the displayed sets. Because the AUC of parameter set 2 only differs in the third decimal place from parameter set 1 but requires 43% less runtime, it is preferred over set 1 and represents the “accuracy-optimized” setting. As parameter set 7 is the fastest parameter set with nonetheless high AUC and EFs, we selected it as the “runtime-optimized” setting.

### Runtime Analysis

Runtime calculations were performed for the *RunTimeSet* on a PC equipped with an Intel i5-9500 (3.0 GHz) processor, 32 GB of main memory, and both a Toshiba KBG40ZNS512G solid-state drive (SSD, 512 GB, model NVMe) and a Hitachi HUA722020ALA330 hard-disk drive (HDD, 2 TB) with an xfs file system. The PostgreSQL database used for PiMine was initialized and run on the same computer. PostgreSQL was initialized on either the HDD or SSD. The PostgreSQL parameters are listed in the Supporting Information (Tables S1 and S2). Runtimes were measured using the Linux command line tool “/usr/bin/time” (wall clock time).

We do not list any runtimes of the SQLite database because these are about three times longer. We do not recommend using this database type for large databases, as SQLite performs searching using only one thread, while the PostgreSQL database potentially uses multiple available threads of the employed processor. Also, while SQLite works out of the box for PiMine, a PostgreSQL database is also easy to set up and use.

## Results and Discussion

### Current Datasets for the Evaluation of Protein-Protein Interface Comparison Methods and an Unbiased Alternative

**Figure 2:**
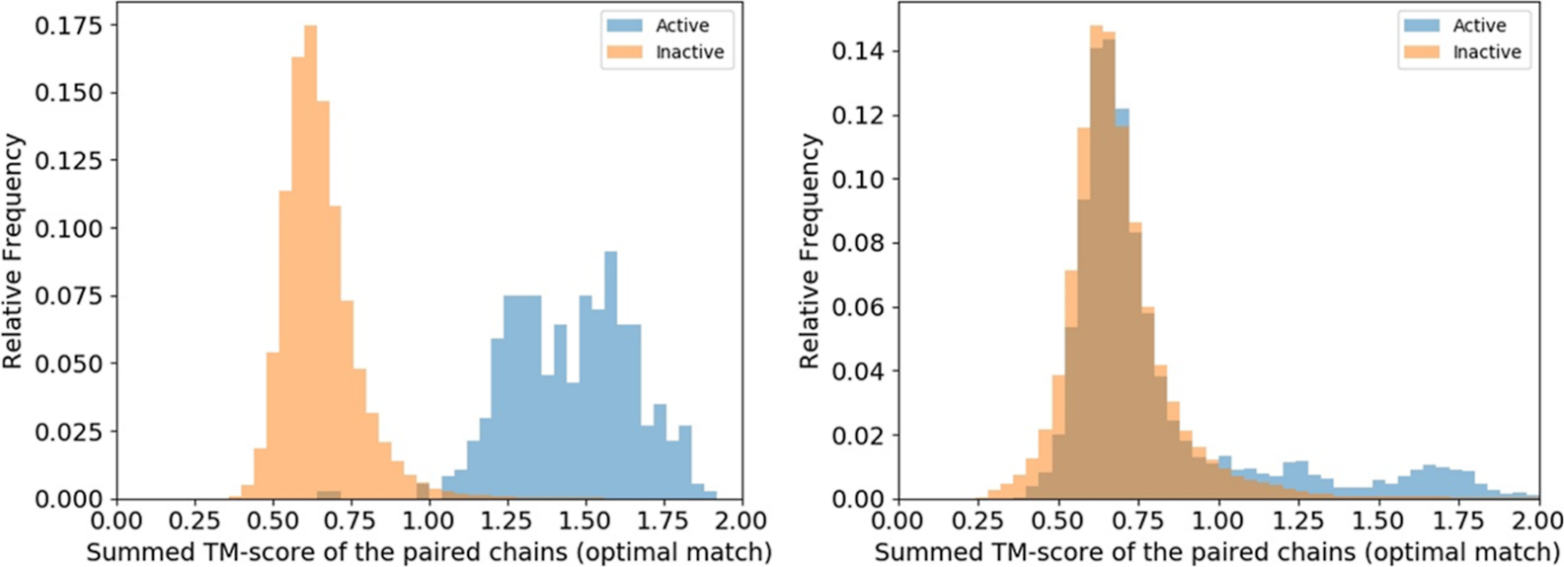
Global similarity of the interface chains in the similar and dissimilar protein-protein interface pairs of the *Dimer597* (left) and the *Keskin* (right) set. TM-align^18^ was applied to compare the chains of the protein-protein complex pairs. For the chains A and B of the first protein-protein complex and the chains A′ and B′ of the second protein-protein complex, we can find two potential pairings (AA′ and BB′ or AB′ and BA′). The chain pairing with the highest sum of TM-scores for both chains was used to generate the histograms. The blue bars indicate the distribution of TM-scores for the dissimilar interfaces (“inactive”), while the orange bars show the distribution of the TM-score for interfaces regarded as similar in the dataset (“active”).

The definition of binding site similarity and dissimilarity depends on the model used.^50^ The model heavily influences the development of corresponding similarity measures, e.g., shape- versus pharmacophore- versus complementarity-based measures, sequence- vs structure-based similarity assessments, or even simple descriptor-based analyses. However, in structure-based modeling, the objective classification of site pairs is rarely undertaken, although it is the only robust way to reliably compare methods with differing underlying similarity measures. An analysis of the currently applied datasets for evaluating interface comparison methods underpins this phenomenon. We summarize the underlying hypotheses for establishing these datasets in the Supporting Information (Paragraph S2). The *Dimer597* set relies on similar SCOP superfamily assignments of the chains forming the interface. Thus, both chains share a similar fold, and structure comparison methods should detect these similarities (Figure 2). Furthermore, the definition of dissimilar interfaces is exclusively based on the SCOP family. Therefore, interface pairs in the dataset might share a high local similarity in terms of pharmacophore and shape properties. The authors tried to avoid biologically irrelevant interfaces due to crystal packing by scoring the interaction energy. In contrast, potential crystal artifacts were excluded based on distances in the *Keskin* set. This dataset relies on the assumption that similar interfaces should share a similar geometrical arrangement of α-carbon atoms, identical interface residues, and similar size.

The key research question for tools that detect similar protein interfaces is the identification of potential binding partners. As indicated above, fast global protein comparison tools such as FoldSeek^45^ are highly suitable for detecting obvious global similarities to deduce binding partners based on global chain similarity. However, we find cases of interface similarities without global fold similarity, posing a major challenge for developing interface comparison methods.^51^ To overcome the lack of appropriate datasets for such scenarios, we propose a workflow that looks for globally related chains in proteins. However, we consider only protein-protein interface pairs whose second chain pair is globally structurally unrelated and whose interacting chains bind to similar regions. Therefore, we can assume that the globally unrelated chains share common interface properties to enable binding to very similar interfaces. Based on an analysis of interfaces predicted as biologically relevant (see the Methods section for more details), we could identify 77 pairs of proteins of this type. Hiding them in a set of interface pairs where the partner chain pair binds to globally related chains but in different regions enabled us to establish a dataset of similar interfaces that are not biased by global or fold similarity. Therefore, considering only the globally unrelated chain pairs and assessing whether tools can enrich them based on the score will provide us with the most unbiased set of similar interfaces we can achieve.

### Evaluating the Performance Using Both Chains

**Figure 3:**
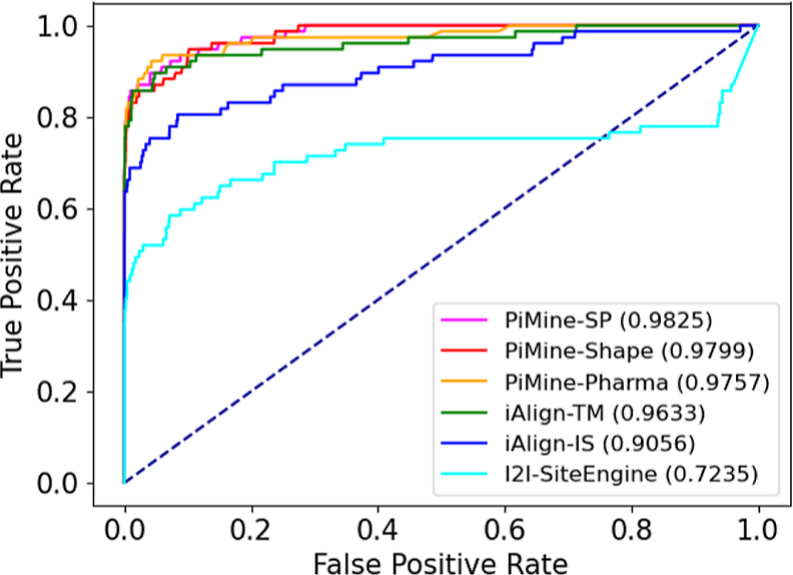
ROC curves for predicting related interfaces using iAlign, I2I-SiteEngine, and PiMine when comparing all interface chain pairs of the *PiMineSet*.

The performance of PiMine regarding the ability to distinguish similar interfaces from less similar ones is compared with one of two existing methods that are among the most cited in this context: iAlign and I2I-SiteEngine. For evaluating I2I-SiteEngine, we use the total score as formerly reported on the web server. For PiMine, we show the results with the runtime-optimized parameters; results using the accuracy-optimized parameters are shown in the Supporting Information (Figures S2–S5). Considering both chains of the interface, the methods show promising early enrichment for the *PiMineSet* (Table 5). The EFs at 0.1, 0.5, and 1% are perfect. From 2% onward, the EFs decrease. In particular, iAlign with the TM-score and PiMine, irrespective of the scoring function, manage to correctly predict similar interfaces in the top-ranked pairs, while I2I-SiteEngine is considerably worse at higher percentages. Again, from 2% onward, iAlign’s IS-score performs worse than its TM-score, with EFs on average lower by 0.12. The ROC curve reflects this trend (Figure 3). With an AUC of approximately 0.98, all scoring functions of PiMine lead to convincing results. iAlign achieves a similar AUC to PiMine with the TM-score. This good performance is likely due to the presence of sequentially related chains that are easy to detect by this sequence-dependent method. Using the IS-score leads to a significantly lower AUC. Thus, the contact overlap factor of iAlign’s IS-score reduces the accuracy. Intriguingly, iAlign performs even poorer for sequence-independent calculations (Figure S6), indicating that this setting is not necessarily beneficial for retrieving both-remote interface similarities and sequence-dependent relationships. I2I-SiteEngine has the lowest AUC (0.72) and early enrichment. After an increase up to TPR 0.7, the slope of the ROC curve of I2I-SiteEngine decreases only slightly and the curve drops below the baseline from a TPR of 0.75. I2I-SiteEngine builds triangles for both single-chain protein interfaces of the protein-protein interface and does not focus on only one side. This approach usually considers interactions between chains but, in this case, prevents the enrichment of similar interfaces in the top-ranked interface pairs.

**Table 5:** Normalized Enrichment Factors (EFs) of the Three Methods iAlign, I2I-SiteEngine, and PiMine with Their Respective Scoring Functions on the *PiMineSet*

EF at	iAlign		I2I-SiteEngine	PiMine		
	TM-score	IS-score		SP-score	pharma score	shape score
0.1%	1.0	1.0	1.0	1.0	1.0	1.0
0.5%	1.0	1.0	1.0	1.0	1.0	1.0
1.0%	1.0	1.0	1.0	1.0	1.0	1.0
2.0%	0.98	0.89	0.62	0.96	0.96	0.98
5.0%	0.86	0.73	0.52	0.87	0.88	0.86
10.0%	0.91	0.78	0.58	0.92	0.94	0.88
20.0%	0.94	0.83	0.66	0.96	0.96	0.96

### Evaluating the Performance Using a Single Chain

**Figure 4:**
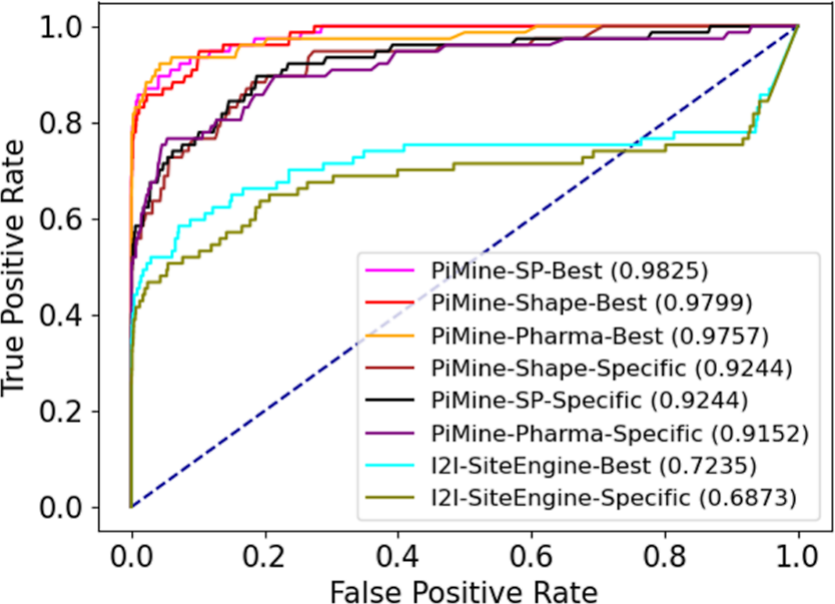
ROC curves for predicting related interfaces using PiMine and I2I-SiteEngine on the *PiMineSet*. Both the highest scoring results called “best” and the ones displaying the interface similarity between the dissimilar chains (“specific”) are shown.

To evaluate the performance in binding partner detection, we took all complexes of the *PiMineSet* and removed the sequentially similar protein chains from the complexes for scoring. The second chain of the complex was only used for defining the interface residues of the query chain and was not considered in the comparison steps. Thus, only the sequentially and structurally unrelated chains with a TM-score below 0.5 were compared (Table 6 and Figure 4). iAlign requires two chains for the definition of an interface. A definition of interface residues of only one chain is not feasible with the publicly available tool of iAlign. Therefore, this tool could not be benchmarked accordingly. As expected, the early enrichment of PiMine and I2I-SiteEngine is lower than before. I2I-SiteEngine does not achieve a perfect EF from 1% onward, but overall, the EFs decrease by approximately 6% on average compared with the results for considering the related chains as well. PiMine’s EFs decrease by 14.75, 17.5, and 17.75% on average for the SP, pharma, and shape scoring functions, respectively. Except for the EFs at 5% dataset coverage, all EF values are above 0.7, indicating promising early enrichment for binding partner detection. As users will usually investigate only a tiny fraction of the best-scored matches, they can expect a high percentage of meaningful similarities in these results. All scoring functions of PiMine lead to an AUC of approximately 0.92. On average, these AUC values are about 0.06 lower than when also comparing the highly similar chains. I2I-SiteEngine reaches an AUC of 0.69. The difference in the AUC when including similar chains is only 0.03. When excluding similar chains, PiMine performs worse but still gives convincing results. The early enrichment is still higher than I2I-SiteEngine’s, with a TPR of 0.55 versus about 0.42. Even above this TPR, the ROC curves rise significantly, showing that PiMine can still distinguish between related and unrelated interface pairs, even without using chains with high sequence and structure similarity.

**Table 6:** Normalized enrichment factors (EFs) of I2I-SiteEngine and PiMine on the *PiMineSet* When Evaluating Interface Similarities Only between Dissimilar Chains

EF at	I2I-SiteEngine	PiMine		
		SP-score	pharma score	shape score
0.1%	1.0	1.0	1.0	1.0
0.5%	1.0	1.0	1.0	1.0
1.0%	0.89	1.0	1.0	1.0
2.0%	0.58	0.78	0.73	0.75
5.0%	0.47	0.68	0.68	0.64
10.0%	0.52	0.75	0.77	0.74
20.0%	0.62	0.87	0.86	0.88

### Alignment Performance

**Figure 5:**
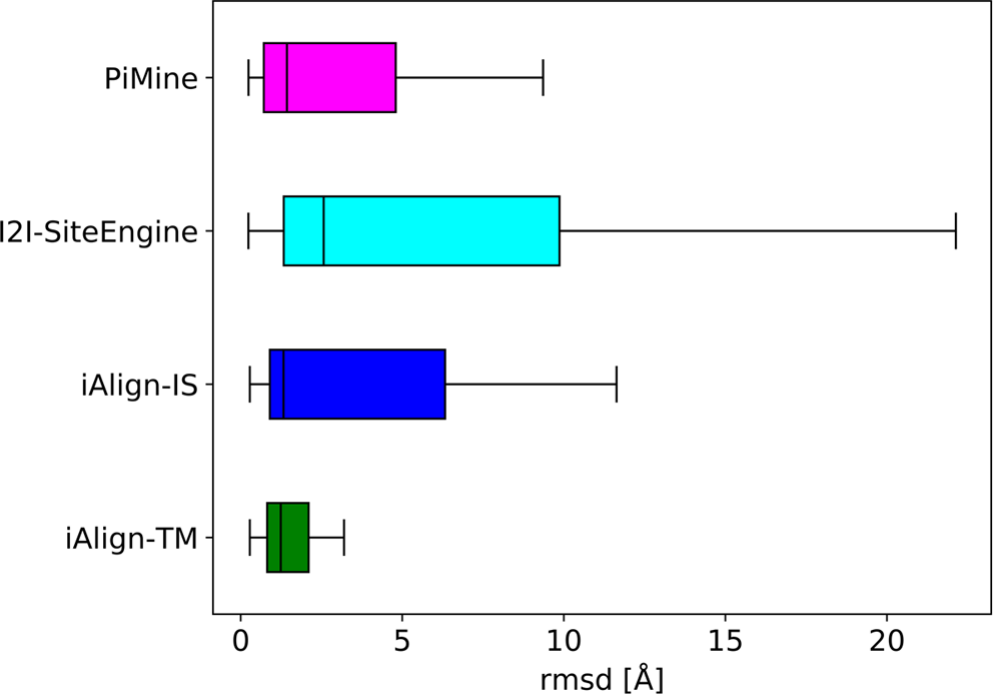
Box plots showing the rmsd distributions for the alignments of the active pairs of the *PiMineSet*. The rmsd values of ten pairs not aligned by I2I-SiteEngine are missing in the corresponding plot.

Due to the nature of the *PiMineSet*, we can also prepare “correct” alignments based on the sequentially and structurally related chains of both protein-protein complexes. These alignments were obtained using TM-align.^19^ Accordingly, we can evaluate the ability of the interface comparison methods to produce the correct alignments. To this end, we calculated the rmsd between the unrelated protein chains of the active interface pairs for alignments of the three methods under investigation. For homodimeric structures, we generated both possible alignments and selected the minimum rmsd for the chains of both alignments. Figure 5 shows the results of this analysis. The overall quality of the I2I-SiteEngine alignments with a median of 2.57 Å, but an upper quartile reaching up to 10 Å is poor. In contrast, iAlign performs well with medians of 1.32 and 1.23 Å for the IS-score and TM-score, respectively. This finding is expectable considering that iAlign compares complete chains, calculates all four possible pairwise chain alignments, and chooses the one with the highest score. Correspondingly, it nearly perfectly reproduces the TM-align-based alignments. As discussed above, we could not test whether aligning the structurally more dissimilar chain pairs might lead to similar results. PiMine shows an equally good performance with a median of 1.43 Å. Considering that the method only compares interface residues, we consider its alignment accuracy as convincing.

### Benchmarking Protein-Protein Interface Comparison Methods on Earlier Datasets

In the first paragraph, we highlighted the drawbacks of earlier datasets. However, as users might want to focus on a distinct model of interface similarity, we also evaluated PiMine for these datasets in the following.

#### The *Dimer597* Set

**Figure 6:**
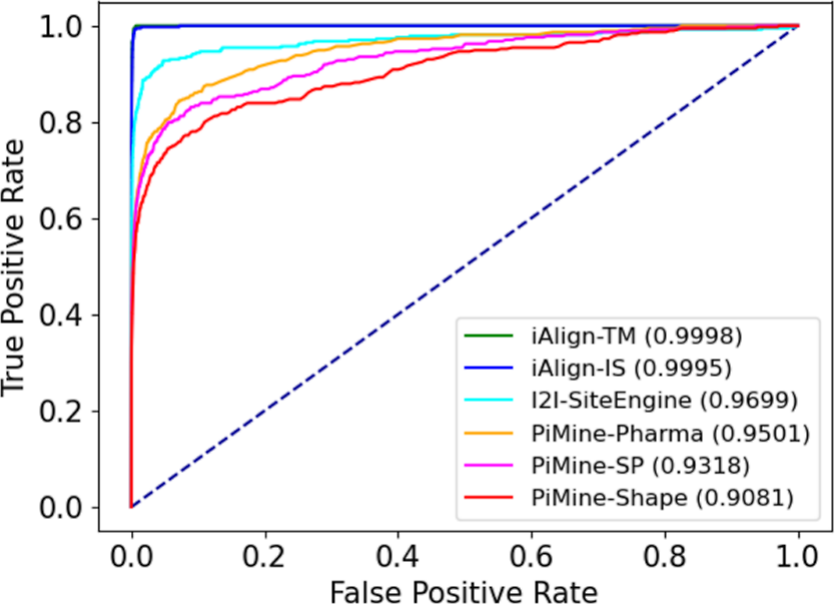
ROC curves for predicting related interfaces using three methods, iAlign, I2I-SiteEngine, and PiMine, to compare the interface pairs of the *Dimer597* set.

Figure 6 shows the results for the *Dimer597* set as ROC curves. Here, our structure-driven method performs worse than iAlign with a focus on the protein sequence and slightly worse than I2I-SiteEngine with AUCs of approximately 0.95 for the pharma score (orange), 0.93 for the SP-score (pink), and 0.91 for the shape score (red). Notably, the performance of iAlign with an AUC of nearly 1 is superior for this dataset. The dataset was created to assess the function of iAlign. The authors used TM-align, which strongly resembles iAlign in the algorithmic approach. The latter and the usage of whole protein chains by iAlign explain the nearly perfect AUC and EFs. The PiMine pharma score performs best compared with the worst-performing shape score. The SP-score combines pharma and shape scores, which leads to an AUC of over 0.93, generally indicating the comparatively good performance of a method. In a real-life scenario of screening an interface database, we would expect the methods scoring similar pairs highest and would only consider a tiny fraction of the whole dataset. Therefore, we also analyzed the early enrichment. At a percentage of 0.1% or 168 actives out of 177 pairs of the dataset, iAlign achieves a normalized EF of 0.97 and 0.95 for the TM-score and IS-score, respectively. I2I-SiteEngine’s-normalized EF is at 0.86, while the ones for PiMine are 0.72 (SP), 0.72 (pharma), and 0.71 (shape). The main difference between PiMine and the other tools is the scoring method. While I2I-SiteEngine calculates the sum of the similarities of both interface chains and iAlign also considers both chains forming the interface, the PiMine score constitutes the maximum similarity between a pair of chains of both interfaces. When considering both chains of the corresponding interfaces for alignment selection and scoring, PiMine achieves an EF of 0.91 using the pharma score (Table S3). The corresponding ROC curves are depicted in Figures S7 and S8. Nevertheless, we decided against this setting for PiMine, which is specifically designed for single-chain-based applications such as binding partner prediction.

**Figure 7:**
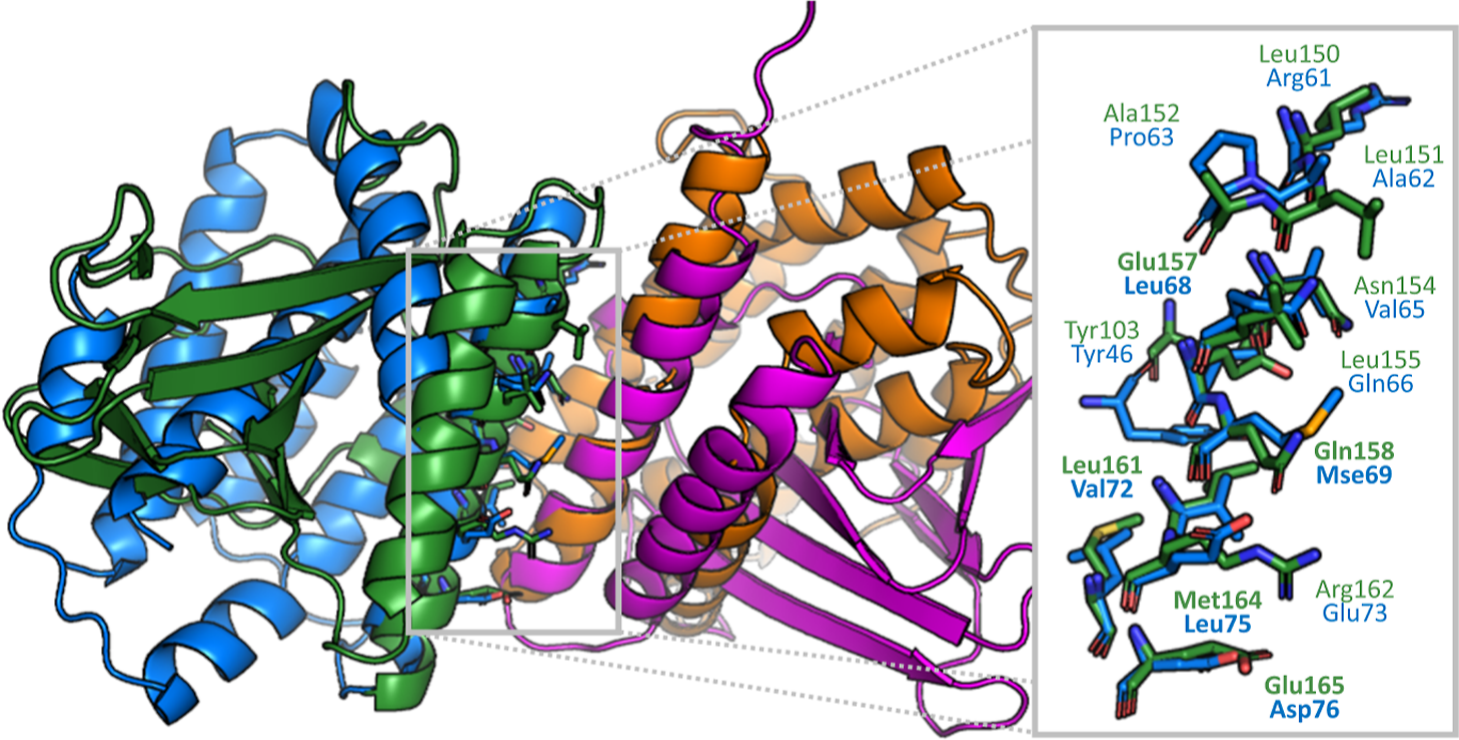
Alignment of the protein-protein interfaces of chains D (green) and C (magenta) of the PDB entry 1xja and chains B (purple) and A (orange) of entry 1wwm. This match represents a *Dimer597* set pair classified as dissimilar. Matching interface residues are shown on the right. Residues in bold represent residues whose α-carbon atoms were used to generate the best-scoring tetrahedron filter. Molecular graphics generated with the PyMOL(TM) Molecular Graphics System, version 2.3.^53^

Overall, iAlign and I2I-SiteEngine reliably predict similarities between the interfaces of highly related proteins. However, there might be a significant number of false negatives in the dataset, as it relies on the assumption that chains with low overall structural similarity do not have similar interfaces. However, this does not necessarily hold, as shown in various studies.^51,52^ One of these potential false negatives is the detected interface similarity of the structures with PDB code 1xja (interface between chains C and D) and 1wwm (interface between chains A and B) (Figure 7). The score calculated by PiMine for this protein-protein interface is ranked 49th for the 177,248 pairwise comparisons of the *Dimer597* set. The alignment indicates a similarity of the two single-chain interfaces not captured in the dataset.

Thus, even though our method appears inferior when applied to this dataset, it was developed with a focus on similar interfaces without explicit sequence information and finding nonobvious similarities not detectable based on overall structural similarity. This feature might lead to the high false positive rate of our method, as the dataset does not account for such remote relationships.

#### The *Keskin* Set

**Figure 8:**
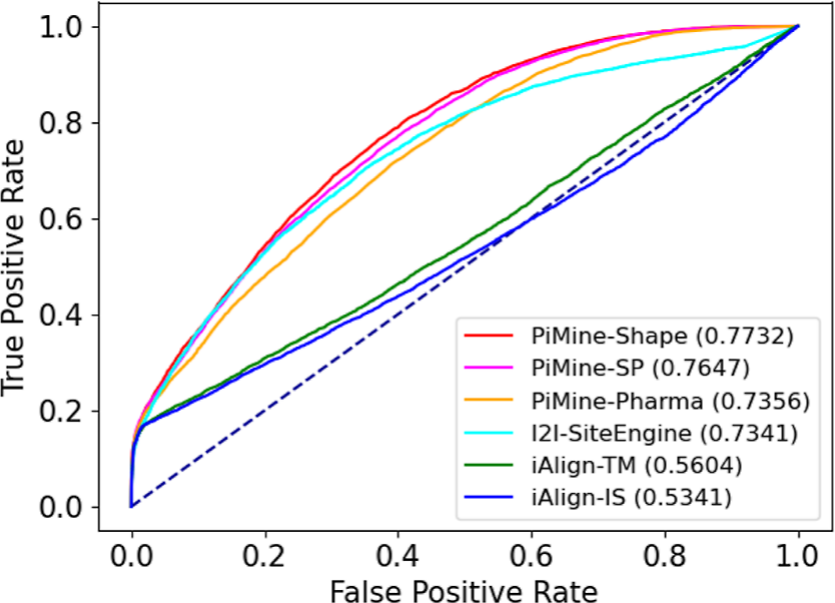
ROC curves for predicting related interfaces using three methods, iAlign, I2I-SiteEngine, and PiMine, to compare the interface pairs of the *Keskin* set.

For this dataset, PiMine performs comparably to I2I-SiteEngine (Figure 8) and it achieves the best results with the shape and SP-scores. The pharmacophore-based score of PiMine performs worst with an AUC of 0.74, while I2I-SiteEngine achieves an AUC of 0.73. In contrast, iAlign performs significantly worse. If using the sequence-independent setting, the AUC increases slightly, but still, the method performs considerably poorer than PiMine and I2I-SiteEngine (Figure S9). The early enrichment of the three methods (Table 7) shows that all methods perform well at 0.1 and 0.5%. PiMine performs best and achieves EFs of up to 0.93 or 0.63 in contrast to iAlign with up to 0.86 or 0.56 and I2I-SiteEngine with 0.86 or 0.51 at these percentages.

**Table 7:** Normalized EFs at 0.1 and 0.5% of the Three Methods iAlign, I2I-SiteEngine, and PiMine with Their Respective Scoring Functions on the *Keskin* Set

EF at	iAlign-TM	iAlign-IS	I2I-SiteEngine	PiMine		
	TM-score	IS-score		SP-score	pharma score	shape score
0.1%	0.86	0.82	0.86	0.93	0.93	0.91
0.5%	0.56	0.54	0.51	0.63	0.62	0.61

**Figure 9:**
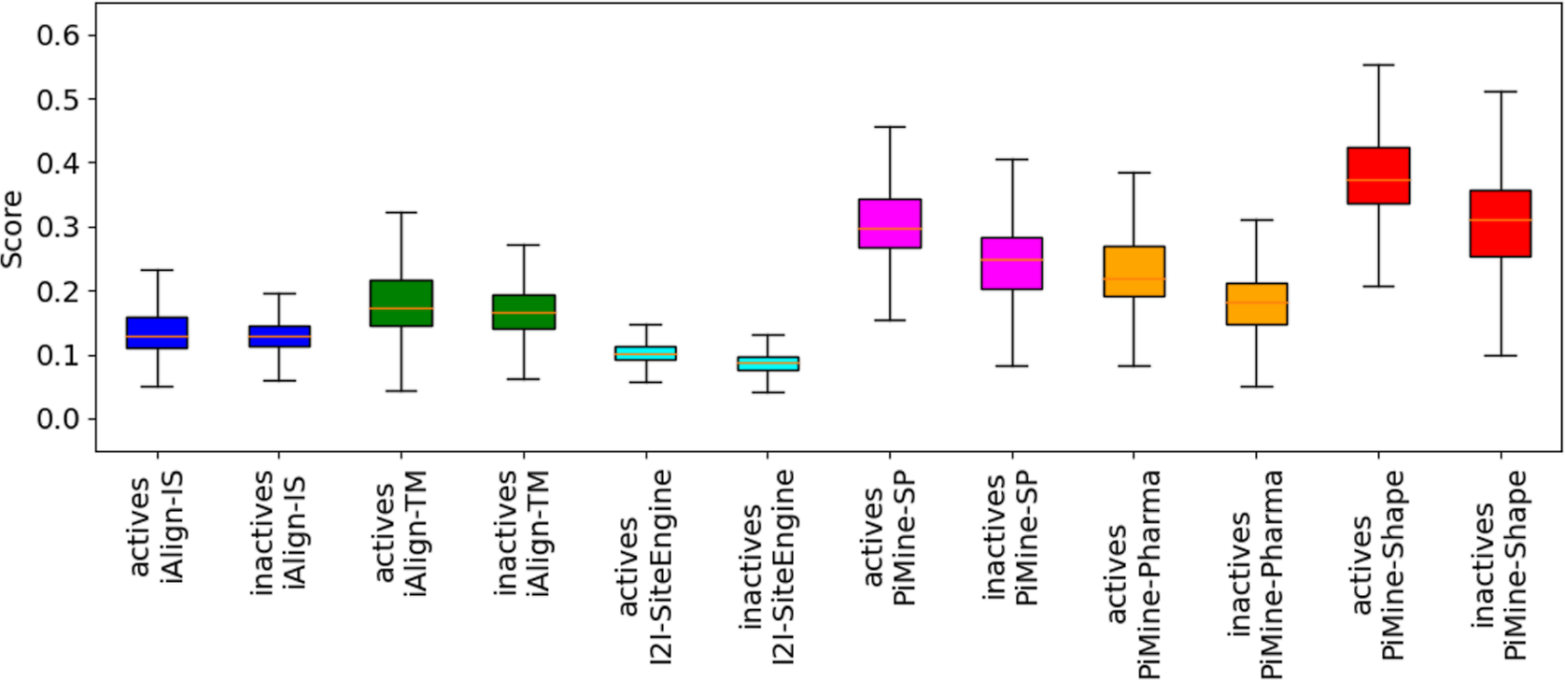
Box plots showing the score distributions of actives and inactives for the *Keskin* set using three methods, iAlign, I2I-SiteEngine, and PiMine. Scores are normalized between zero and one. For a better overview, outliers are omitted.

Starting from a true positive rate of about 0.19, iAlign’s ROC curve increases slowly. At TPRs between 0.6 and 1.0, the IS-score decreases below the random baseline. This poor performance of iAlign may result from the lack of sequential similarities, as iAlign relies on global structure similarities (see the Introduction). Up to an FPR of 0.58, I2I-SiteEngine performs at a level comparable to the shape and SP-score of PiMine. After that, the I2I-SiteEngine’s TPR drops below the one of PiMine. The ROC curves for the shape and SP-scores of PiMine are nearly indistinguishable. While the pharma score achieves a comparable early enrichment, the AUC is considerably lower. To further analyze how well the scores of the methods differentiate between actives and inactives, we normalized the scores between zero and one and created box plots (Figure 9). A plot containing the outliers considering the complete score range can be found in Supporting Information (Figure S10). According to the box plots, the score distributions of the actives and inactives overlap for each method. For iAlign, however, the interquartile range overlaps nearly completely. The median scores for the similar and dissimilar pairs are nearly identical, leading to the low AUC of iAlign. Compared to the *Dimer597* set, iAlign scores for the actives in the *Keskin* set are much lower (Figure S11) and similar to the scores of the inactive pairs in the *Dimer597* set. In contrast, I2I-SiteEngine and PiMine distinguish well between similar and dissimilar interfaces. Of the PiMine scores, the shape score leads to the highest AUC values. These results suggest a high structural similarity between the interface pairs despite the low sequence similarity and a high degree of similarity between the interface surfaces. In summary, PiMine and I2I-SiteEngine perform well on this set of sequentially unrelated protein chain pairs with, nevertheless, structurally similar interfaces.

### Runtime

The *RunTimeSet* was used for all subsequent analyses. For the run-time measurements, we randomly generated an index number over all interfaces and picked the interface corresponding to this index to analyze against all others of the dataset. Next, we preprocessed all interfaces with PiMine, iAlign, and I2I-SiteEngine. PiMine created a database comprising 59,803 structures and 169,689 interfaces. Overall, 1300 interfaces of 300 PDB entries were not processed, corresponding to a coverage of 99.8% of the structures and 99.4% of the interfaces. Missing structures can be attributed to very short peptides. PDB entry 1b05, for example, is missing because one chain consists of only three residues. After the database was created, the runtimes for calculating the similarity scores were measured (Table 8). Generally, PiMine is the fastest when using runtime-optimized parameters and a PostgreSQL database. First, we only look at the runtimes using the HDD. The accuracy-optimized parameters are approximately 2.1 times slower than the runtime-optimized ones. The runtime of the I2I-SiteEngine is the longest among the three methods (371.6 h). It is 9.1 and 4.2 times slower than PiMine with the runtime-optimized and accuracy-optimized parameters, respectively. iAlign, with a runtime of 3.9 h, is about 95.3 times faster than I2I-SiteEngine and 10.5 or 22.5 times faster than PiMine with the runtime-optimized and accuracy-optimized parameters, respectively. As comparisons with PiMine and I2I-SiteEngine take multiple days and an up-to-date PC is often equipped with an SSD, we reevaluated the runtimes. Because I2I-SiteEngine uses a data structure requiring about 1.8 TB of storage space, we split the dataset into chunks of 10,000 interfaces and screened them consecutively with the tool. The runtime in Table 8 constitutes the sum of the runtimes for the individual chunks. PiMine performs about 54% better on the SSD than on the HDD. It calculates all similarity scores within 1-2 days, depending on the chosen parameters. PiMine’s database-driven similarity analysis largely relies on short reading times. In contrast, iAlign’s and I2I-SiteEngine’s runtimes on the SSD are similar to the ones on the HDD, with 3.4 h (approximately 13% faster) and 369.6 h (approximately 1% faster), respectively. Therefore, we assume the similarity calculation to be more time-consuming than reading the interfaces. In summary, iAlign is much faster than PiMine and preferable if users intend to screen large databases with evolutionary-related PPIs within minutes. In most other cases, a runtime of 19 h for PiMine with runtime-optimized parameters is acceptable for most use cases.

**Table 8:** Runtime Analysis of PiMine, iAlign, and I2I-SiteEngine Using the *RunTimeSet* (169,944 Comparisons)^a^

method	drive	runtime [h]
PiMine (runtime-optimized)	HDD	41.0
PiMine (accuracy-optimized)		87.7
iAlign		3.9
I2I-SiteEngine		371.6
PiMine (runtime-optimized)	SSD	19.1
PiMine (accuracy-optimized)		39.0
iAlign		3.4
I2I-SiteEngine		369.6

a PiMine’s runtime is assessed with the runtime-optimized and accuracy-optimized parameters using a PostgreSQL database initialized either on a hard-disk drive (HDD) or a solid-state drive (SSD). We measured the runtimes for screening this set for similarities to the randomly chosen interface between chains A and B of PDB entry 3t4m.

### Case Studies

#### Retrospective Application Examples

We finally investigated the applicability of PiMine in practice. To this end, we looked for application studies using the tools, as listed in Table 1. We screened citing articles for the successful applications of the corresponding tools on PubMed (PubMed, Bethesda (MD): National Library of Medicine (US), https://www.ncbi.nlm.nih.gov/pubmed/, last access: November 30th, 2023).

**Figure 10:**
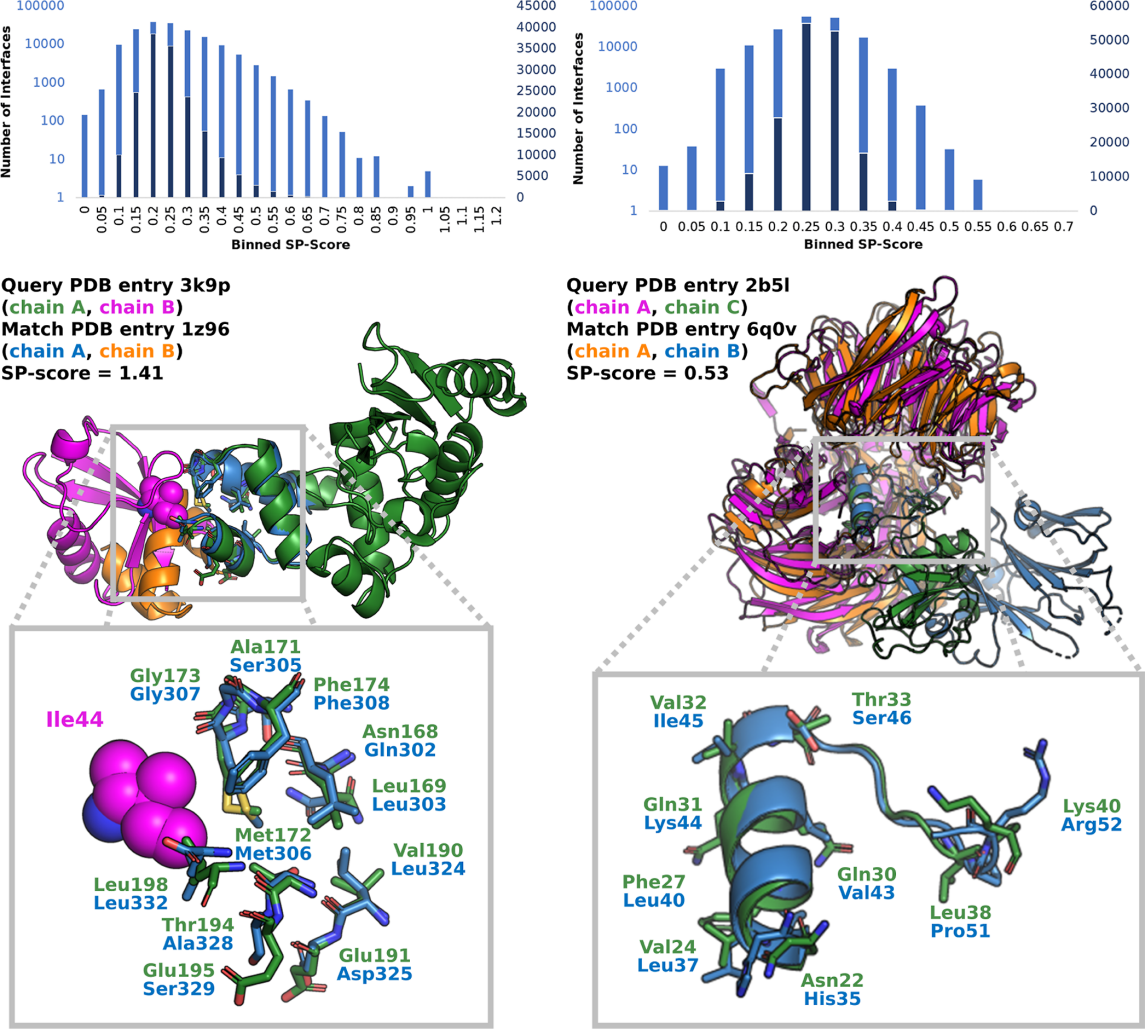
Score distributions and alignments for the retrospective application examples. Published similar protein interfaces were hidden in the *RunTimeSet*. Retrieval of these interfaces with the originally used single-chain query interfaces was analyzed. Score distributions are represented in a logarithmic (blue) and linear (orange) scale. Left: according to a published example by Keren-Kaplan and co-workers,^54^ chain A of PDB entry 3k9p (ubiquitin-conjugating enzyme E2 K) was used to query the dataset. Alignment for one of the three highest-scored true positive matches (chain A of PDB entry 1z96, UBA-domain protein Mud1) is depicted below the corresponding score distribution. Similar residues are labeled in the enlarged depiction at the bottom. Right: according to a published example by Cheng and colleagues,^24^ chain C of PDB entry 2b5l (Simian virus 5 V protein) was used to query the dataset. The alignment with the second highest-scored match (chain B of PDB entry 6q0v, DDB1- and CUL4-associated factor 15) is depicted below the corresponding score distribution. Residues 398 to 713 of chain B of PDB entry 2b5l were omitted for visualization purposes. Similar residues are labeled in the enlarged depiction at the bottom. Molecular graphics generated with the PyMOL(TM) Molecular Graphics System, version 2.3.^53^

Keren-Kaplan and co-workers use the SiteEngine^16^ algorithm to identify ubiquitin-binding domains (UBDs).^54^ To assess the performance of PiMine to also detect the same UBDs in a set of interfaces of sequentially diverse chains, we hid the PDB entries with an interface of two chains (PDB entries 3k9o, 2bwb, 1z96, 3b0f, 2ooa, 3ihp, 2qho, 4ae4, and 1wrd) in our *RunTimeSet* and used the interface of PDB entry 3k9p as query (ubiquitin-conjugating enzyme E2 K in a complex with ubiquitin). The three best-scored hits are known examples of UBDs from the corresponding publication (PDB entries 3k9o, 1z96, and 2qho). A fourth PDB entry (2bwb) was on rank 141 with an SP-score of 0.67 (Figure S12). Figure 10 shows an exemplary alignment of the ubiquitin-conjugating enzyme E2K and the ubiquitin-associated (UBA) domain-containing protein Mud1 (chain A of PDB entry 1z96). Other PDB entries reported by Keren-Kaplan et al. were not significantly high-ranked by PiMine. The PDB entry 2ooa is also a homodimer. Comparing this entry to a structure in complex with ubiquitin (PDB entry 2oob) shows that a reasonable interface was found in the database. Nevertheless, PiMine could not detect any similarity. A visual comparison of the interfaces of PDB entries 3k9p (chain A) and 2ooa (chain B) does not reveal any specific physicochemical similarities besides some similar hydrophobic residues. For chain B of PDB entry 3b0f, a homodimeric structure, we cannot ensure that the interface relevant to the interaction with ubiquitin was stored in the database, as there are no known complexes with ubiquitin. An alignment to the homodimer of PDB entry 2ooa shows that the screened interface does not correspond to the ubiquitin-binding interface. The interface region between ubiquitin and chain A of PDB entry 3ihp is much larger than the one in the query structure, explaining the low rank of this interface (SP-score = 0.19). For PDB entry 4ae4, we cannot ensure having stored the correct interface region interacting with ubiquitin, as the structure is a homodimer and there is no known structure in a complex with ubiquitin. The SP-score for the interface of PDB entry 1wrd (SP-score = 0.38) indicates no significant similarities detected by PiMine. However, both interfaces share some common residues. Searching with the interfaces of both chains of the query complex, we find PDB entry 1wrd at rank 11 (interface between chains A and B). Besides the four detected hits, we find several high-scoring matches with uncharacterized proteins, KDPG aldolases from different organisms (e.g., PDB entry 1vhc, *Haemophilus influenzae*), and the Holliday junction ATP-dependent DNA helicase RuvA (e.g., PDB entry 3ik5, *Salmonella enterica*), which is already known for its similarity to other proteins of the CATH superfamily of UBA domains. Keren-Kaplan and co-workers might not have found these similar interfaces, as they restricted their search to PDB chains from eukaryotic organisms. Exemplary alignments are depicted in the Supporting Information (Figure S12) and show convincing similarities in a helix interacting with the recognition patch of ubiquitin with a characteristic Ile44. To the best of our knowledge, these proteins are not annotated as ubiquitin-binding partners. However, the interactions of proteins with ubiquitin are manifold^55^ and highly variable. The high SP-score indicates potentially uncharacterized ubiquitin-binding patches on these proteins. However, we could not verify this finding due to a lack of structures of these proteins in complex with ubiquitin. In summary, we can show that PiMine enriches similar interfaces, although we are missing some of the previously detected interfaces hidden in the dataset.

Cheng and co-workers used their method PCalign to explore known cases of viral mimicry.^24^ Altogether, they presented three case studies. We explored the ability of PiMine to retrieve the described interface similarities in our *RunTimeSet*. To this end, we hid the corresponding similar interfaces to the viral interfaces in this set. The first example is the known interaction of the murid herpesvirus 4 M3 protein, a known chemokine binding protein, with CC chemokine ligand 2 (PDB entry 2nz1), modulating the human immune response.^56^ The aim was to show a similarity toward a homodimer of the C–C motif chemokine 2 (PDB entry 1dok). Screening our dataset with the chain representing the M3 protein, we found this target interface only at rank 8482 with a very low SP-score of 0.44. The first human chemokine dimer pair that matches our single-chain interface is on rank 1013 (PDB entry 5cmd). The best-scored match is a complex between the M3 protein and the C–C motif chemokine 2 (PDB entry 1ml0). Besides, the most significant similarities were found to transcription factors RelA, RelB, nuclear factor NF-κ-B p105 subunit (NFKB1), and nuclear factor NF-κ-B p100 subunit (NFKB2), with SP-scores above 0.7. The score distribution indicates the high relevance of these matches (Supporting Information, Figure S13). However, upon visualizing the corresponding alignments, we only find similarities regarding a single β-strand. The match cannot be used to relate to novel interaction partners as they severely clash in the alignment with the query chain. When using both chains of the interface for screening the dataset, we only find chemokines in the best-scored 50 results, indicating the general applicability of PiMine. However, these hits would probably not have been found by using only the structure of the viral protein. In a second study, Cheng and co-workers analyzed the structure of the Simian virus 5 V protein. Its interface is said to be similar to that of DNA damage-binding protein 2 (DDB2). The latter binds to DDB1, thus participating in UV-induced nucleotide excision repair, and stimulates E2F1-activated transcription. A blockage of this interaction by the V protein is known to support viral pathogenesis. Using chain C of PDB entry 2b5l, representing the interface of the V protein, we screened for the documented similar site of PDB entry 3ei4 (chains A and B) hidden in our *RunTimeSet*. Although this protein was only on rank 803, we found a complex of DDB1 and DDB1- and CUL4-associated factor 15 (DCAF15; PDB entry 6q0v, Figure 10) on the second-highest rank. The best-scored match corresponds to the interface of a dimer of 6-deoxyerythronolide-B synthase EryA2, modules 3 and 4 (PDB entry 1pzq), and seems to be a false positive match. This hypothesis could be verified by visually inspecting the detected similarity. Only a single query helix overlaps with the found interface. More importantly, there are several clashes among the corresponding interface chains. Also, the ratio of the pharma and shape scores is low, indicating insufficient validity of the match. Intriguingly, other complexes of DDB1 and DDB2 were also found on very low ranks with PiMine. Using both chains, we find a complex of DDB1 and DDB2 at rank 6 (PDB entry 4a0l). The example of this complex found by Cheng and co-workers (PDB entry 3ei4) is on rank 28. Analyzing the alignments using only the viral chain and both interface chains as queries, we only find minor differences and see that an N-terminal helix as a crucial part of the interface is the main similarity. The best-scored hit, however, is a complex of DDB1 and the DNA excision repair protein ERCC-8 (PDB entry 4a11). Again, we find the N-terminal helix as the most striking similarity between the viral protein and the DDB1 binding partner. This helix was reported earlier as the crucial viral motif for mimicking DDB1 binding partners.^57^ Depictions of all discussed alignments and score distributions can be found in the Supporting Information (Figure S14). Another example of viral mimicry is the interaction of Hendra virus glycoprotein G (PDB entry 2vsk, chain A) with ephrin-B2. Similarities of the viral glycoprotein and the cognate human cell–surface receptor (Eph) are known.^58^ Using the viral glycoprotein interface (only chain A) to search for a known complex of EphB4 and ephrin-B2 (PDB entry 2hle) hidden in our *RunTimeSet*, we found various matches with glycoprotein complexes of other viruses (Nipah virus, e.g., PDB entry 2vsm, Ghanaian henipavirus, e.g., PDB entry 4uf7, Cedar henipavirus, e.g., PDB entry 6p7y, Cedar virus, e.g., PDB entry 6thg) in the best-scored 17 hits. However, the hidden interface is only on rank 19,382. A complex of an ephrin and its receptor is on rank 157 (PDB entry 1kgy): ephrin-B2 in a complex with ephrin type-B receptor 2. Using both interface chains of the query, this match is on rank 20, while PDB entry 4bkf, representing a complex of ephrin-B3 and ephrin type-A receptor 4, is on rank 9, with scores similar to the ones obtained for viral glycoprotein-ephrin complexes. We can explore the structural similarities between the human receptor proteins and the viral glycoproteins by visualizing the corresponding alignments (Supporting Information, Figure S15). The similar interface detected by Cheng and co-workers is still on the lower ranks of the hit list with a low SP-score. A flexible loop region of ephrin proteins is mainly responsible for interactions with other proteins (GH loop). This loop interacts with the DE loop of the receptor proteins and a highly flexible JK loop.^59^ Interactions between these loops are conformationally variable^60^ and might explain why the enrichment and alignment of the viral protein to the similar host interface is challenging for PPI search tools like PiMine.

Altogether, these examples highlight that PiMine retrieves host partners of viral proteins. A visual inspection considering score distributions, the ratio of pharma to shape score, and an analysis of the alignments generated by PiMine enables the user to find truly valid hits. The corresponding alignments show that PiMine not only enriches true positive hits but also provides reliable alignments, often even in the absence of the corresponding binding partner interface. Most intriguingly, our method finds reliable hits even after omitting the globally similar chains of the interfaces.

#### Showcase Study

**Figure 11:**
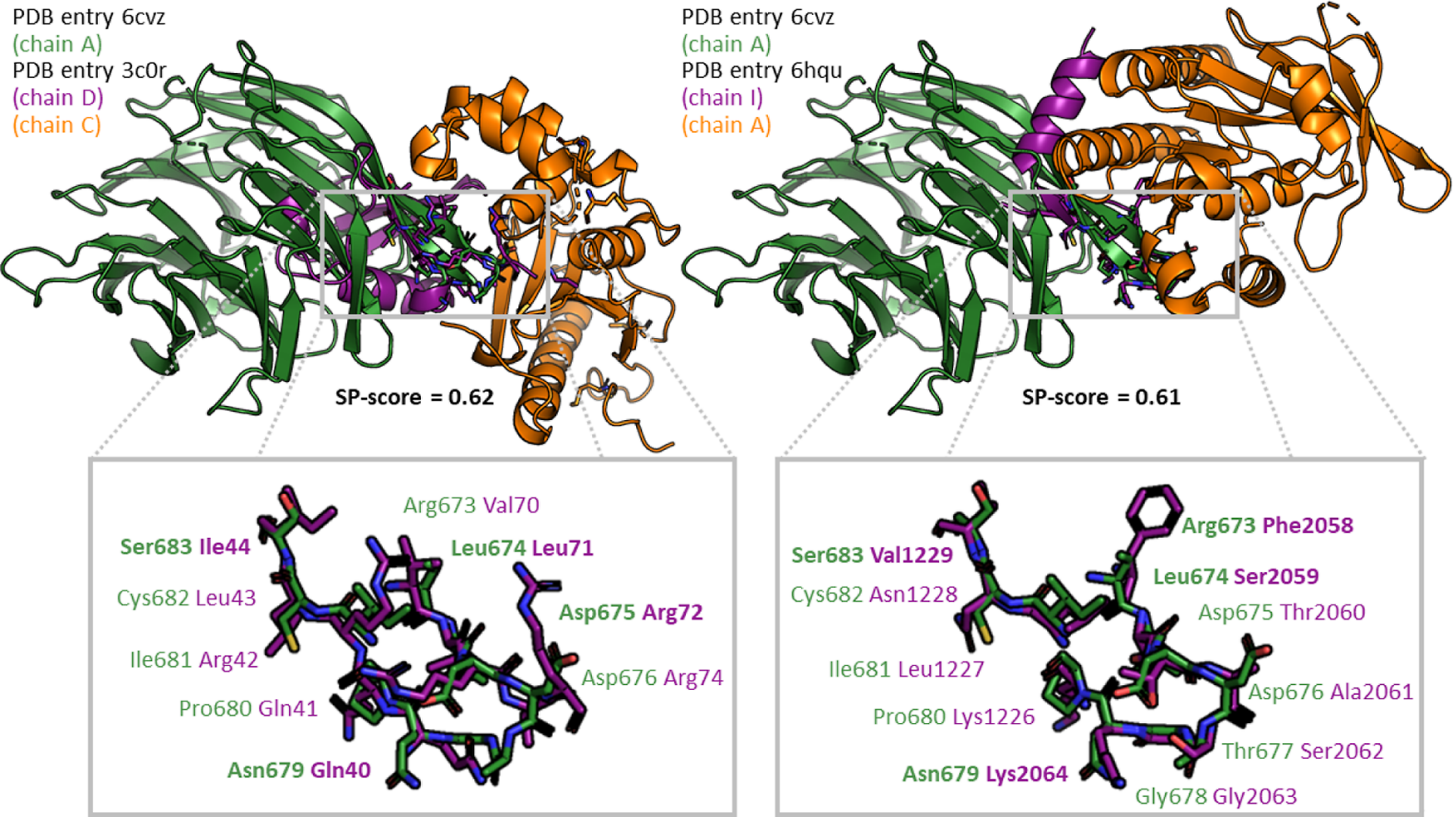
Predicting interaction partners and the structure of the corresponding protein-protein complexes with PiMine. PiMine-based alignment of the protein-protein interfaces of the SPPIDER-predicted interface of chain A (green) of the E3 ubiquitin-protein ligase RFWD3 (PDB entry 6cvz) and chains D (purple, human ubiquitin) and C (orange) of entry 3c0r (left), and chains I (purple) and A (orange, DNA repair and recombination protein RadA from *Pyrococcus furiosus* DSM 3638) of PDB entry 6hqu (right). Matching interface residues are shown below the alignments of the complete proteins. Residues in bold represent residues whose α-carbon atoms were used to generate the best-scoring tetrahedron filter. Molecular graphics generated with the PyMOL(TM) Molecular Graphics System, version 2.3.^53^

To demonstrate the applicability of our novel tool to reveal previously unknown similarities, we investigated whether we could identify potential protein-protein complexes for structurally known proteins with low sequence similarity to other proteins in the PDB. We looked for proteins with known interaction partners but no known structure of the complex in the PDB. One example is the structure of human E3 ubiquitin-protein ligase RFWD3 (PDB entry 6cvz). To date, sequentially related homologues with a sequence similarity of at least 30% cannot be found in the PDB. However, the protein interacts with Rad51, thereby mediating its ubiquitinylation and removal from DNA damage sites,^61^ which, in consequence, enables homologous replication. RFWD3 variants cause Fanconi anemia, complementation group W (FANCW) causing anemia, leukopenia, and thrombopenia,^62^ indicating the impact of the knowledge of the structure of its interactions with other proteins. We predicted the interfaces of RFWD3 using the web server SPPIDER^63^ for protein chain A of PDB entry 6cvz. Upon looking for residues that are in close contact, creating a continuous interface, we chose residues Val570, Glu578, Val580, Gln582, Met622, Asp623, Trp627, Val630, Arg673, Leu674, Asp675, Asp676, Thr677, Gly678, Asn679, Ile681, Ser683, and Gln685 for a PiMine search in protein complexes predicted as biologically relevant (*RunTimeSet*). Unfortunately, comparing PiMine to iAlign and I2I-SiteEngine was infeasible, as both methods rely on a known complex structure between two chains. The alignments of the two highest-scored matches are presented in Figure 11. The score table for this PiMine search is provided in the repository.^48^ We emphasize that PiMine did not find any false positive hits in the top-scored 30 hits of the three benchmark sets used for evaluating the method. Therefore, a user might not expect too many false positives in the highest-scored matches. However, a visualization of the alignments and the score distribution, both parts of the output of PiMine, will help to learn more about the detected similarities. Also, the user should take a look at the highest scores. As seen and discussed earlier, looking at the score distribution might help to learn whether there are any significant similarities to consider.

The best-scored hit of this search was a match to the interface of chains C and D of PDB entry 3c0r (SP-score = 0.62). The interface of our query chain was aligned with the interface of ubiquitin (chain D) in this complex of human ubiquitin and the ubiquitin thioesterase OTU1. This enzyme, to the best of our knowledge, was never predicted as an interaction partner of our query protein. Therefore, we cannot evaluate the validity of this hit. However, a ubiquitin-like interaction with a target protein does not seem to correspond to the annotated function of the protein. Nevertheless, a striking similarity is evident. The significant difference between this score and the average score is depicted in Figure S16 in the Supporting Information and shows a considerable similarity compared with most other interfaces in the dataset. One also finds striking similarities to the so-called four-strand barrelizing versions of the β-grasp fold.^64^ Although the helical structure in this type of protein is missing in the structure of RFWD3, the structure is characterized by two strands forming a conserved insert in this type of protein fold, which is also the region aligned to ubiquitin by PiMine.

The second-best hit (SP-score = 0.61), however, is the humanized RadA mutant HumRadA22 from *Pyrococcus furiosus* DSM 3638 in complex with breast cancer type 2 susceptibility protein (BRCA2), which potentiates recombinational DNA repair (PDB entry 6hqu, interface between chains A and I). In the PiMine alignment, the two β-strands of the latter interaction partner are nicely superposed to the two β-strands of RFWD3 (Figure 11). Based on the alignment, we find that a complex between RFWD3 and HumRadA22 might well form in this way without sterical clashes. One can hypothesize that this predicted interaction might be relevant to the activity of RFWD3 as a ubiquitin-protein ligase, enabling further structure-based research. In summary, this application example shows the benefit of PiMine enabling comparisons of predicted interfaces for comparisons to protein-protein interfaces and the suitability of PiMine to provide hints to the potential structure of an interface of two predicted interacting proteins.

## Conclusions

We presented PiMine, a sequence-independent structural similarity calculation and alignment method for protein-protein interfaces. PiMine aims to detect similarities between the interfaces of evolutionary unrelated protein chain pairs. We have shown that it finds similar protein-protein interfaces in the complete PDB within a single day and is considerably faster than I2I-SiteEngine. iAlign, which is much faster than both methods, reliably detects similarities between evolutionary-related complexes, while it performs weaker than PiMine and I2I-SiteEngine for sequentially unrelated but similar interface pairs. PiMine’s ability to assess the individual scores for single interface pairs, avoiding the necessity of two chains defining an interface for comparison, renders it a valuable tool for predicting the structure of protein-protein complexes, identifying unknown partners of protein chains, or finding potential small molecule-binders of interfaces. Furthermore, PiMine is the method of choice if only one partner of a protein-protein interface is available to search for potentially interacting proteins. We could validate the usability of PiMine based on retrospective application analyses and a predictive case study. For screening settings, where only a low percentage of highest-scoring hits is analyzed, we recommend using the runtime-optimized PiMine parameters. However, if users are interested in specific similarities and a reliable classification of all interface pairs under investigation, e.g., for interface clustering, they can use PiMine’s accuracy mode. The possibility of using single-chain interfaces predicted with external programs to screen a database of structurally characterized protein-protein interfaces is unique for the tool. Based on the score distributions, users get a good estimate of outstanding similarities. A future improvement might involve a statistical measure of the significance of a match. As it is currently possible to search only in known protein-protein interfaces, we intend to extend the search space to global protein surfaces in the future. Following this, an automated clash detection procedure between the individual chains might help to quickly eliminate false positive hits without the need for user intervention. In summary, we presented PiMine as a novel and reliable tool to compare and align protein-protein interfaces. We hope its capabilities and features will assist in broadening our structural and functional understanding of PPIs.

## Data Availability

PiMine is available online as part of the NAOMI ChemBio Suite (https://uhh.de/naomi), which is free for academic use and licensed for commercial use. All datasets and the similarity scores calculated by PiMine, iAlign, and I2I-SiteEngine are available at 10.25592/uhhfdm.13227.

## Supporting Information Available

examples for similar and dissimilar interfaces in the *PiMineSet*; ROC curves for predicting related interfaces using the methods iAlign, I2I-SiteEngine, and PiMine (accuracy-optimized parameters) on the *Dimer597* set; ROC curves for predicting related interfaces using the methods iAlign, I2I-SiteEngine, and PiMine (accuracy-optimized parameters) on the *Keskin* set; ROC curves for predicting related interfaces using the methods iAlign, I2I-SiteEngine, and PiMine (accuracy-optimized parameters) on the *PiMineSet*; ROC curves for predicting similar interfaces of sequentially and structurally similar chains using I2I-SiteEngine and PiMine (accuracy-optimized parameters) on the *PiMineSet* excluding the interfaces of related single chains; ROC curves for predicting related interfaces using the methods iAlign, I2I-SiteEngine, and PiMine (runtime-optimized parameters) on the *PiMineSet*; ROC curves for predicting related interfaces using the methods, iAlign, I2I-SiteEngine, and PiMine (runtime-optimized parameters and scoring using both interfaces of the PPIs) on the *Dimer597* set; ROC curves for predicting related interfaces using the methods iAlign, I2I-SiteEngine, and PiMine (accuracy-optimized parameters and scoring using both interfaces of the PPIs) on the *Dimer597* set; ROC curves for predicting related interfaces using the methods iAlign, I2I-SiteEngine, and PiMine (runtime-optimized parameters) on the *Keskin* set; box plots showing the score distributions of actives (similar interface pairs) and inactives (dissimilar interface pairs) including the outliers for the *Keskin* set using the methods iAlign, I2I-SiteEngine, and PiMine (runtime-optimized parameters); box plots showing the score distributions of actives (similar interface pairs) and inactives (dissimilar interface pairs) for the *Dimer597* set using the methods iAlign, I2I-SiteEngine, and PiMine (runtime-optimized parameters); matches found with a PiMine search for chain A of PDB entry 3k9p (ubiquitin-conjugating enzyme E2-K in complex with ubiquitin); matches found with a PiMine search for chain A of PDB entry 2nz1 (M3 protein of murid herpesvirus 4 in a complex with C–C motif chemokine 2) and interface chains A (green) and D (magenta); matches found with a PiMine search for chain C of PDB entry 2b5l (Simian virus 5 nonstructural protein V in a complex with damage-specific DNA-binding protein (1) and interface chains C (green) and A (magenta); matches found with a PiMine search for chain A of PDB entry 2vsk (Henda virus attachment protein glycoprotein G in a complex with ephrin-B2) and interface chains A (green) and B (magenta); score distribution for the PiMine search for similar interfaces to a predicted one of PDB entry 6cvz (human E3 ubiquitin-protein ligase RFWD3) in the *RunTimeSet*; PostgreSQL 14.6 custom parameters for PiMine runs on the SSD (on the *RunTimeSet*); PostgreSQL 14.6 custom parameters for PiMine runs on the HDD (on the *RunTimeSet*); normalized enrichment factors of the methods iAlign, I2I-SiteEngine, and PiMine (scoring using both interfaces of the PPIs) on the *Dimer597* set; PiMine interface input; and external benchmark datasets for protein-protein interface comparisons (PDF)

## Abbreviations

AUC: area under the ROC curve
EF: enrichment factor
HDD: hard-disk drive
PPI: protein-protein interaction
rmsd: root-mean-square deviation
ROC: receiver operating characteristics
SSD: solid-state drive
