# Supplementary material for: Database-Driven Identification of Structurally Similar Protein-Protein Interfaces
Source: J Chem Inf Model. 2024 Mar 12;64(8):3332–49. doi: 10.1021/acs.jcim.3c01462 (PMC11040719; doi:10.1021/acs.jcim.3c01462)
Supplement: Supplementary file 1 — ci3c01462_si_001.pdf [file ci3c01462_si_001.pdf]

Supporting Information

Database-Driven Identification of Structurally Similar Protein-Protein Interfaces

Joel Graef, Christiane Ehrt*, Thorben Reim, Matthias Rarey*

Universität Hamburg, ZBH - Center for Bioinformatics, Albert-Einstein-Ring 8-10, 22761 Hamburg, Germany

E-mail: christiane.ehrt@uni-hamburg.de, matthias.rarey@uni-hamburg.de

Table of Contents

Figure S1 .... S2
Figure S2 .... S3
Figure S3 .... S3
Figure S4 .... S4
Figure S5 .... S4
Figure S6 .... S5
Figure S7 .... S5
Figure S8 .... S6
Figure S9 .... S6
Figure S10 .... S7
Figure S11 .... S7
Figure S12 .... S8
Figure S13 .... S9
Figure S14 .... S10
Figure S15 .... S11
Figure S16 .... S12
Table S1 .... S12
Table S2 .... S12
Table S3 .... S13
Paragraph S1 .... S14
Paragraph S2 .... S14
References S15

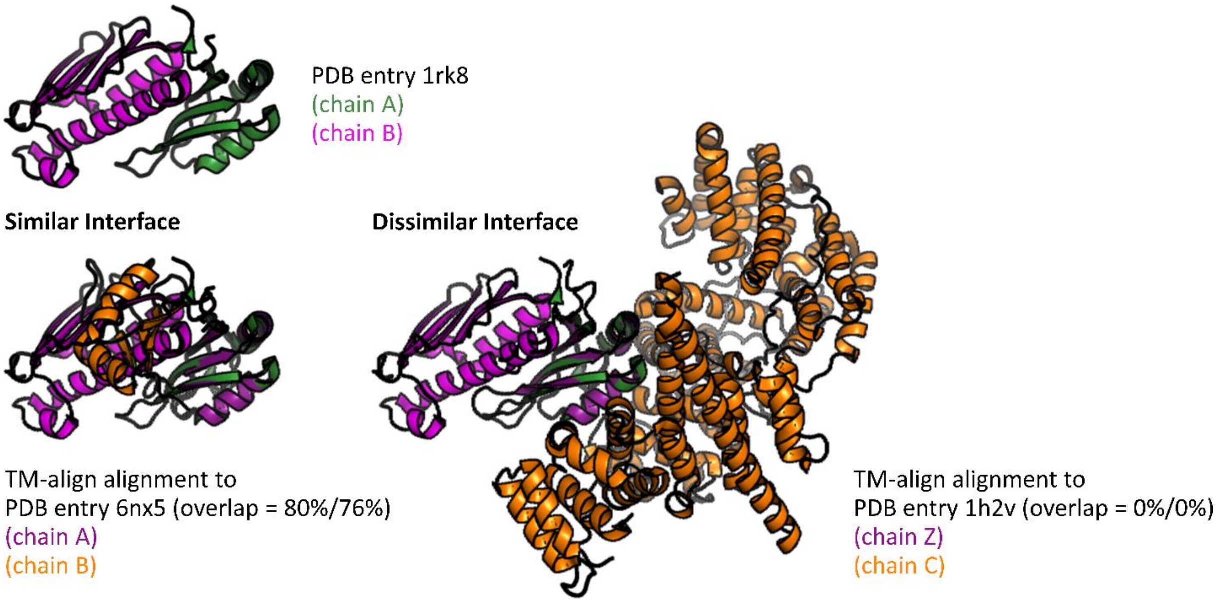

Figure S1. Examples for similar and dissimilar interfaces in the *PiMineSet*. The interface of chains A and B of the query PDB entry 1rk8 is similar to the interface between chains A and B of PDB entry 6nx5. The overlap between the interface residues of the interfaces based on the TM-align alignment is 80% and 76% (left). In contrast, there is no overlap with chains Z and C of PDB entry1h2v. So, the latter interfaces do not have any similarity based on the TM-align alignment (right).

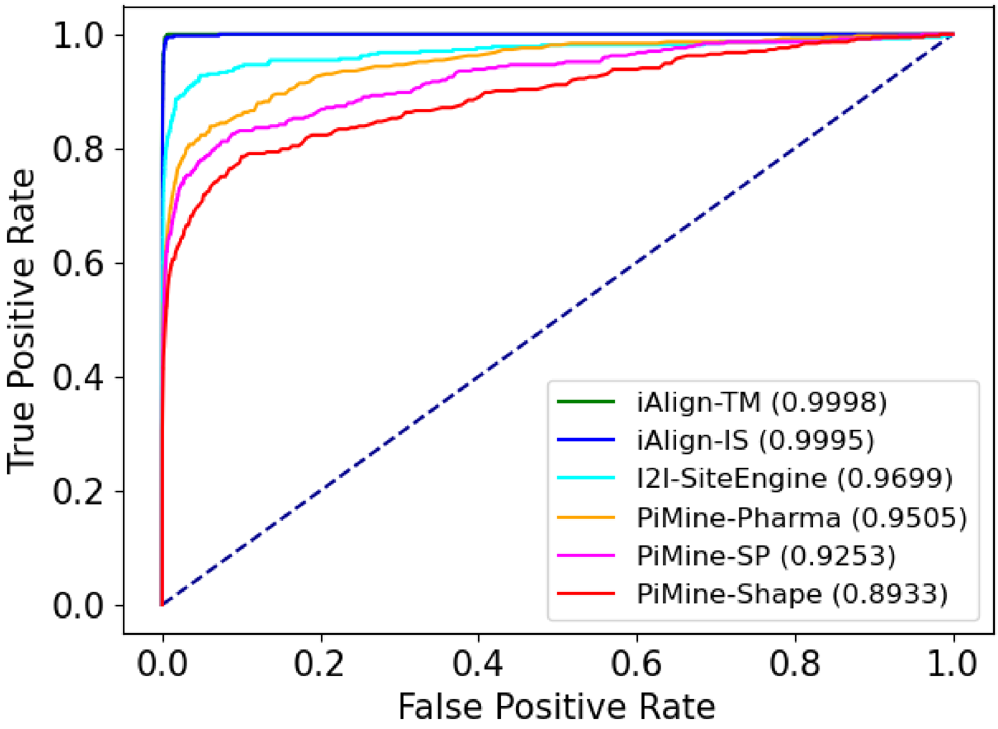

Figure S2. ROC curves for predicting related interfaces using the methods iAlign, I2I-SiteEngine, and PiMine (accuracy-optimized parameters) on the *Dimer597* set.

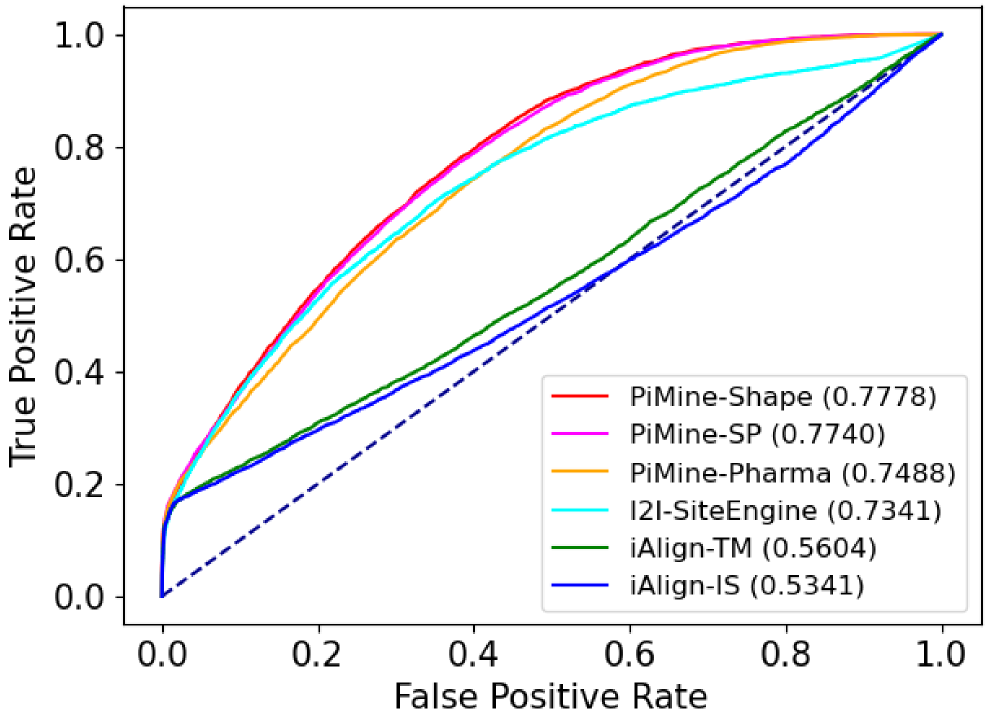

Figure S3. ROC curves for predicting related interfaces using the methods iAlign, I2I-SiteEngine, and PiMine (accuracy-optimized parameters) on the *Keskin* set.

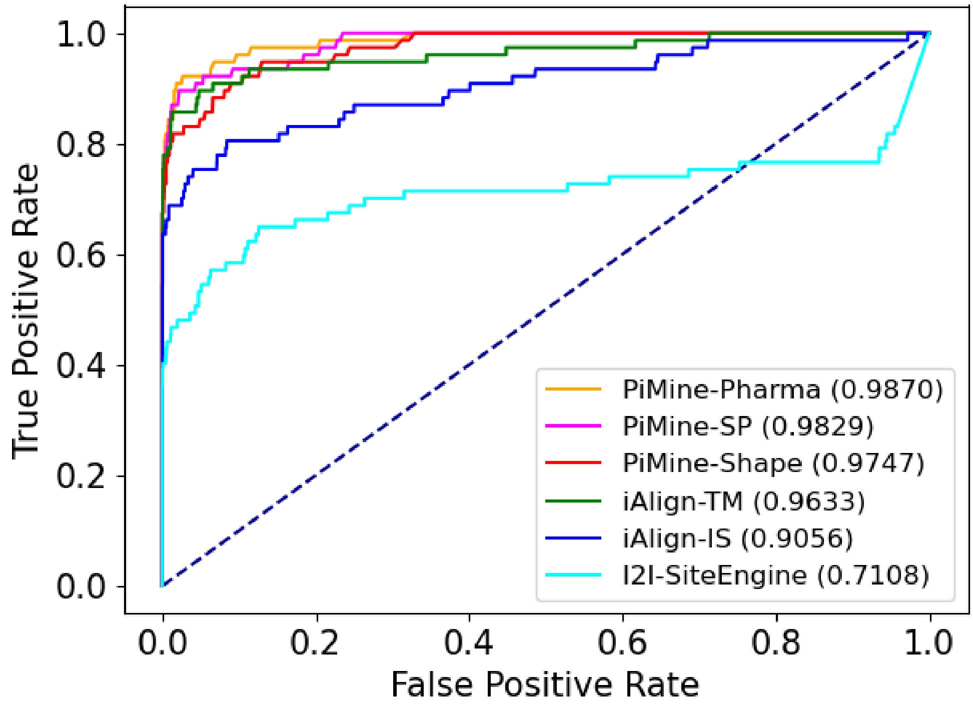

Figure S4. ROC curves for predicting related interfaces using the methods iAlign, I2I-SiteEngine, and PiMine (accuracy-optimized parameters) on the *PiMineSet*.

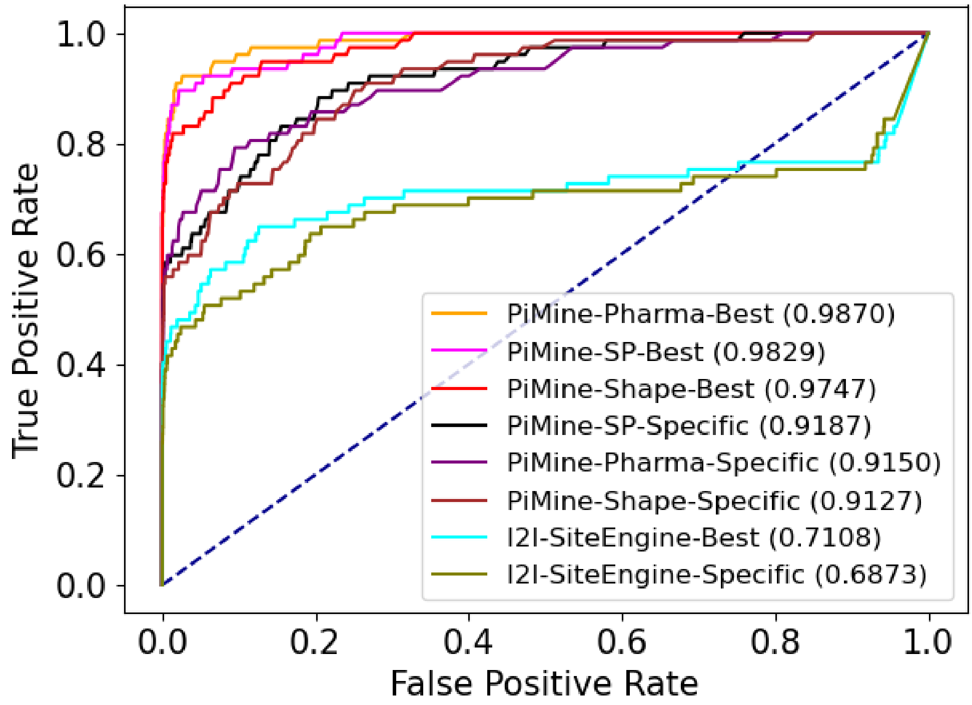

Figure S5. ROC curves for predicting similar interfaces of sequentially and structurally similar chains using I2I-SiteEngine and PiMine (accuracy-optimized parameters) on the *PiMineSet* excluding the interfaces of related single chains. The sequentially and structurally similar chains were excluded from scoring. This scenario is not realizable with iAlign; therefore, the ROC curves for iAlign are missing.

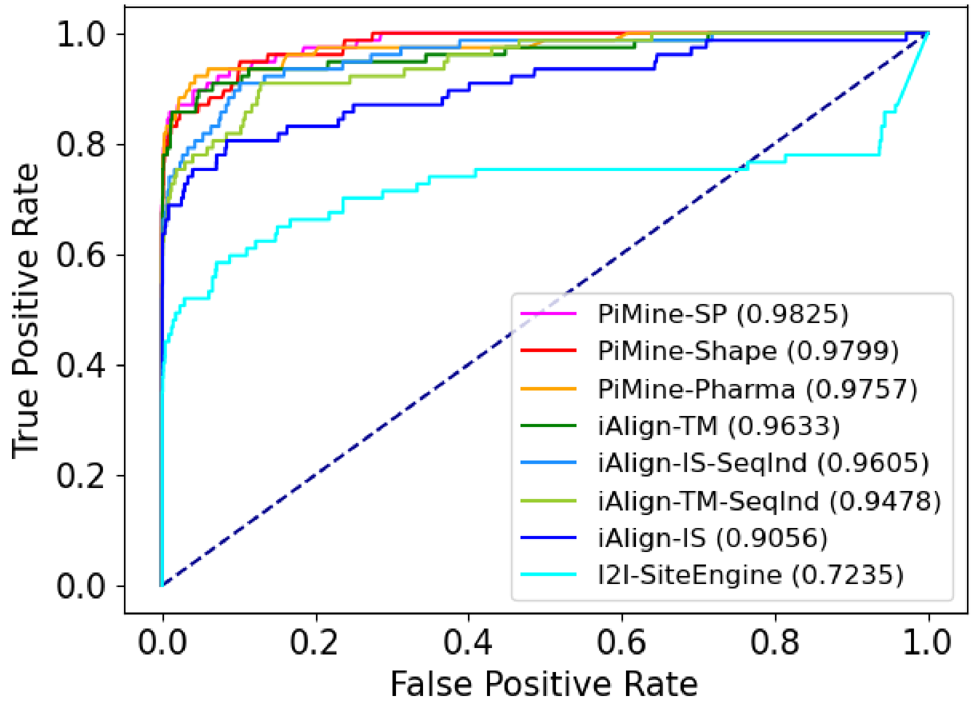

Figure S6. ROC curves for predicting related interfaces using the methods iAlign, I2I-SiteEngine, and PiMine (runtime-optimized parameters) on the *PiMineSet*. The performance of iAlign in sequence-independent (SeqInd) mode is given in light green (TM-score) and light blue (IS-score).

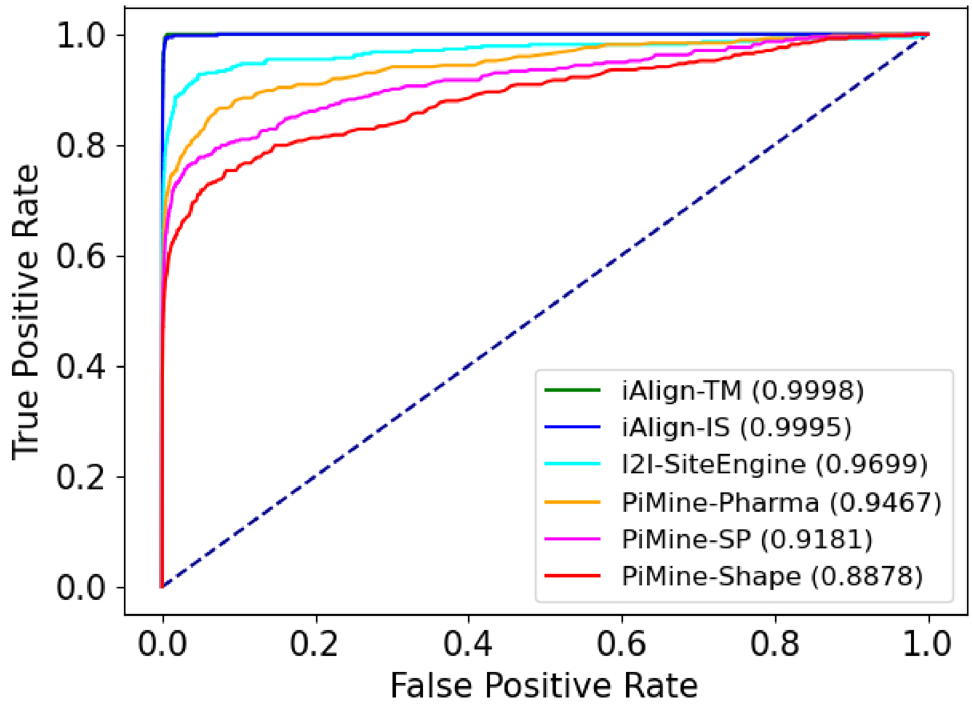

Figure S7. ROC curves for predicting related interfaces using the methods iAlign, I2I-SiteEngine, and PiMine (runtime-optimized parameters and scoring using both interfaces of the PPIs) on the *Dimer597* set.

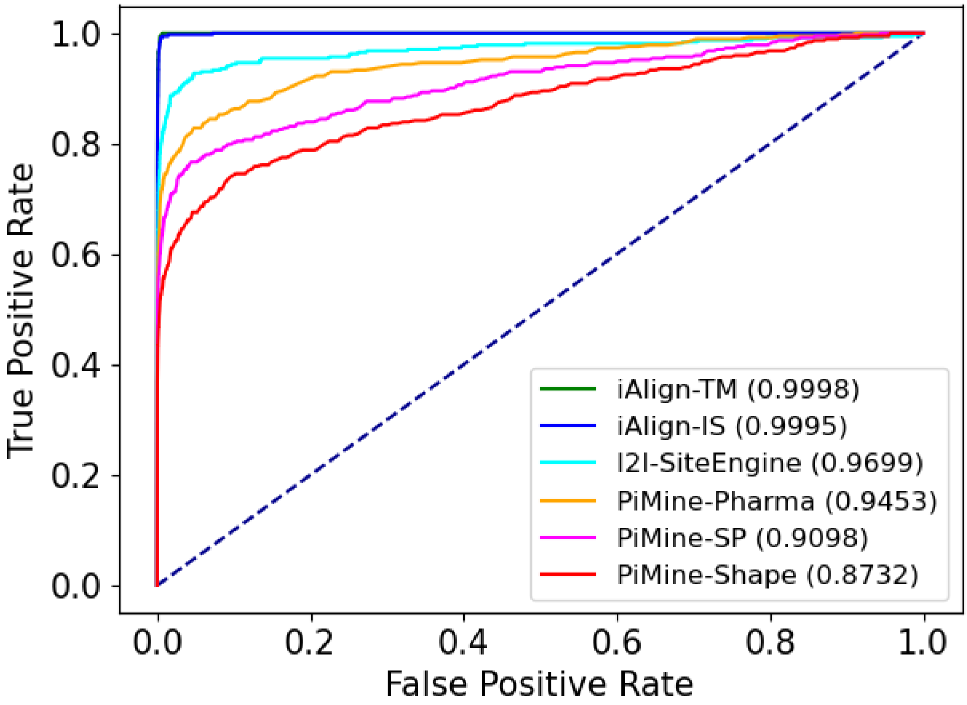

Figure S8. ROC curves for predicting related interfaces using the methods iAlign, I2I-SiteEngine, and PiMine (accuracy-optimized parameters and scoring using both interfaces of the PPIs) on the *Dimer597* set.

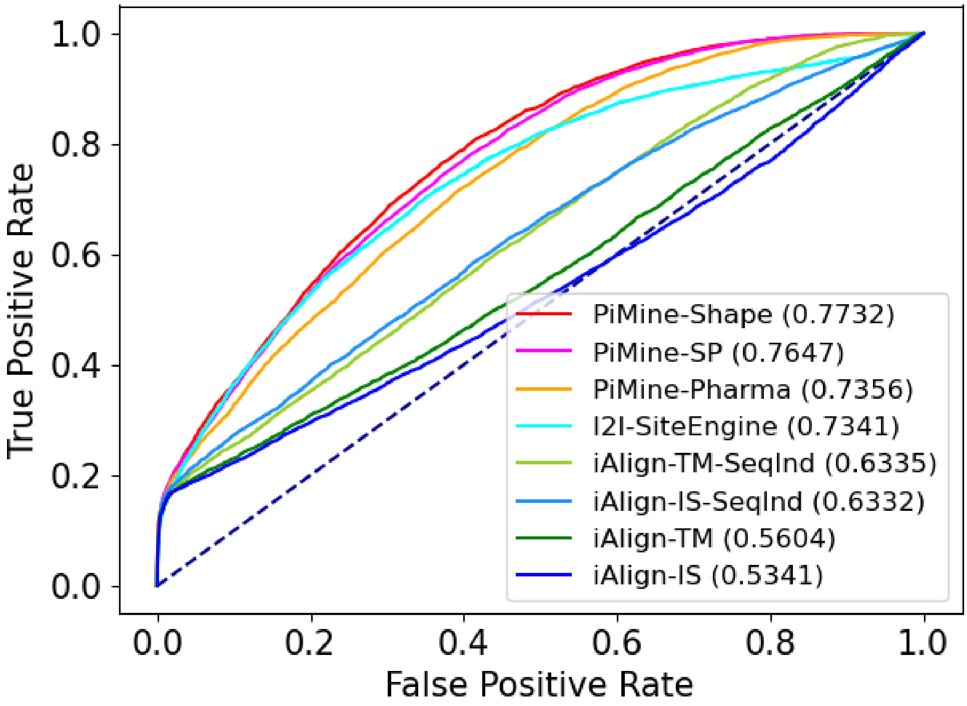

Figure S9. ROC curves for predicting related interfaces using the methods iAlign, I2I-SiteEngine, and PiMine (runtime-optimized parameters) on the *Keskin* set. The performance of iAlign in sequence-independent (SeqInd) mode is given in light green (TM-score) and light blue (IS-score).

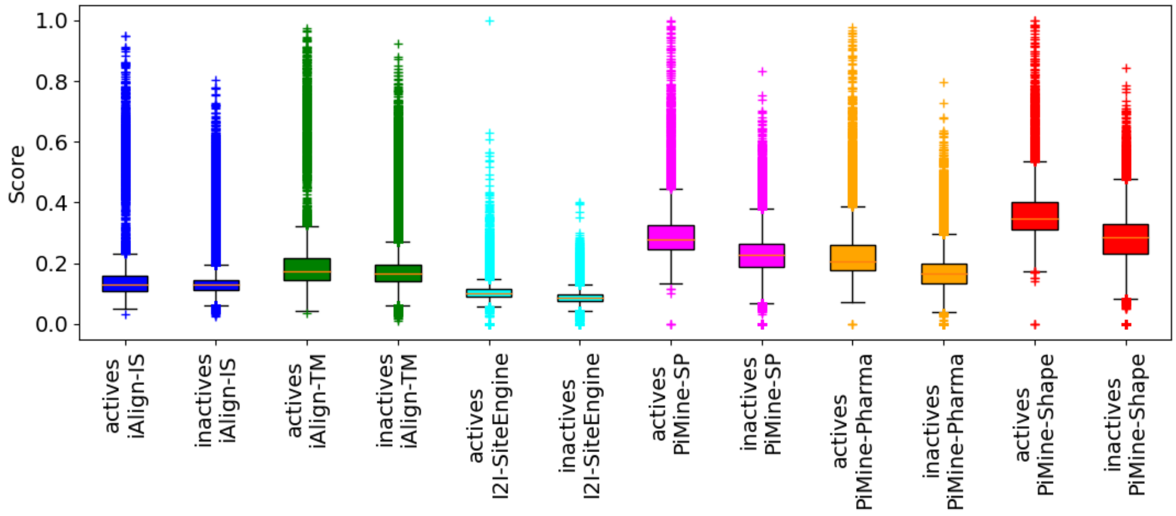

Figure S10. Box plots showing the score distributions of actives (similar interface pairs) and inactives (dissimilar interface pairs) including the outliers for the *Keskin* set using the methods iAlign, I2I-SiteEngine, and PiMine (runtime-optimized parameters).

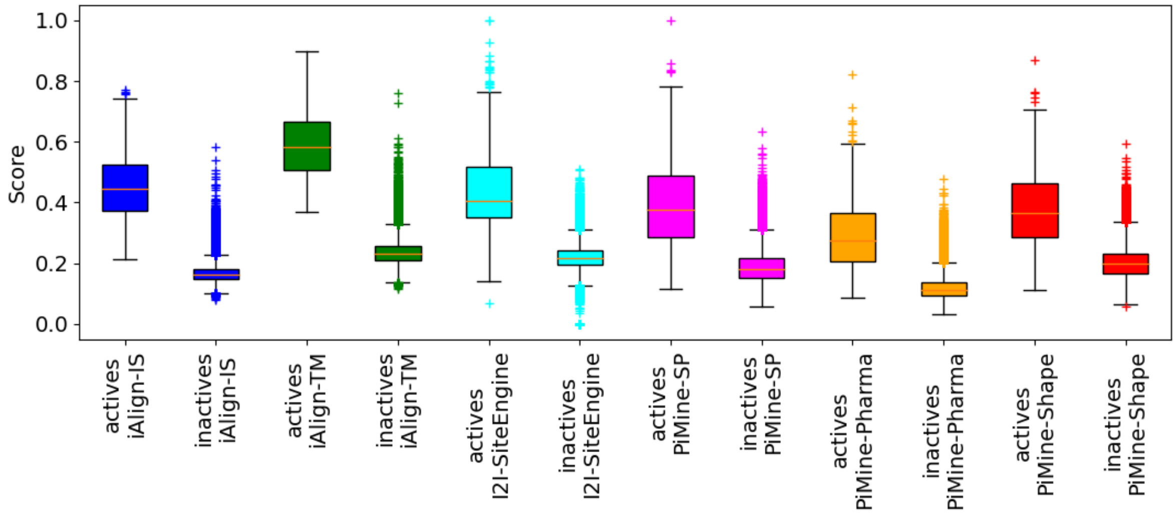

Figure S11. Box plots showing the score distributions of actives (similar interface pairs) and inactives (dissimilar interface pairs) for the *Dimer597* set using the methods iAlign, I2I-SiteEngine, and PiMine (runtime-optimized parameters).

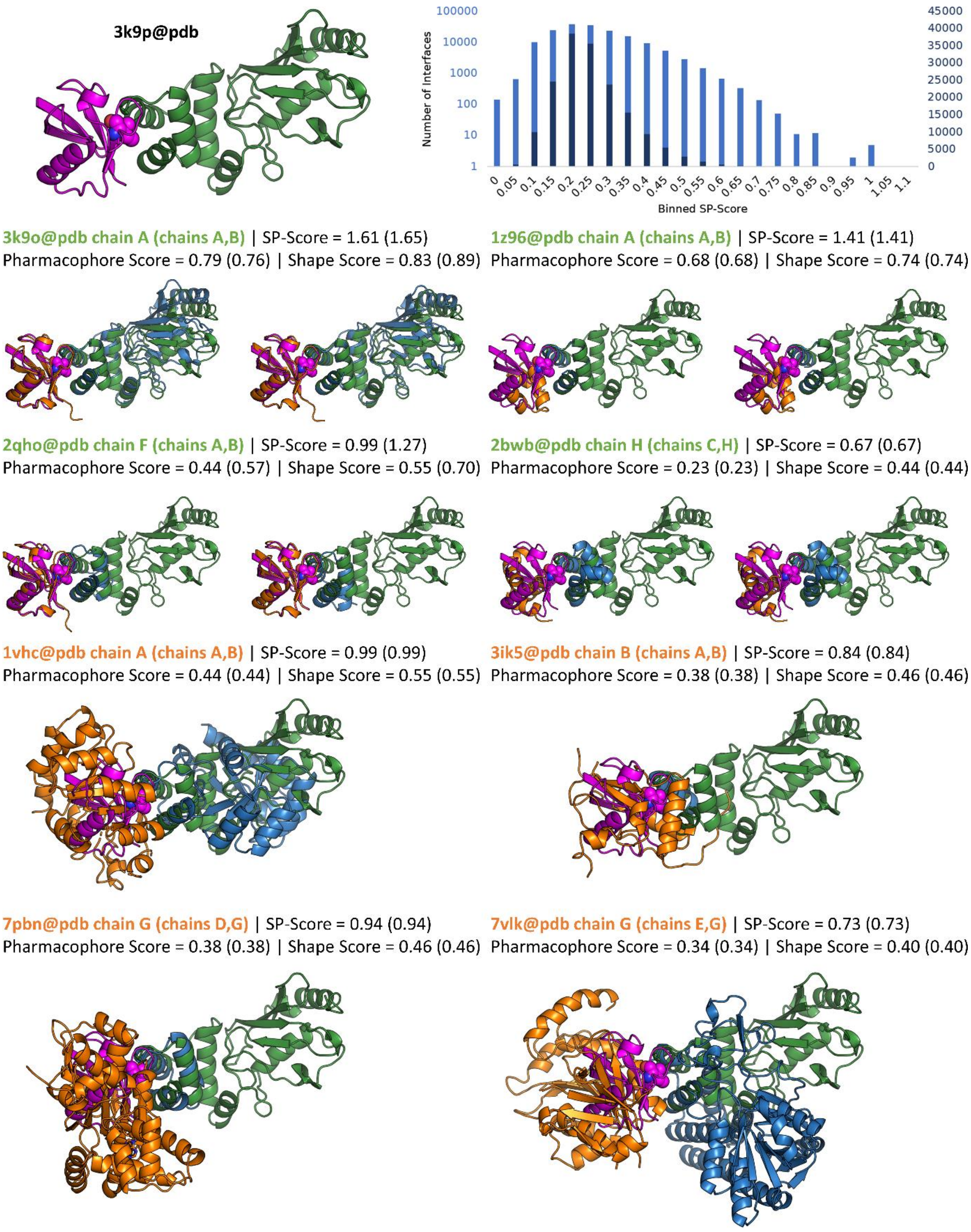

Figure S12. Matches found with a PiMine search for chain A of PDB entry 3k9p (ubiquitin-conjugating enzyme E2-K in complex with ubiquitin). Above, we see the query interface with chain A in green and its binding partner in magenta. On the right, the score distribution for the *RunTimeSet* with the added similar interfaces from the work of Keren-Kaplan and colleagues[1] is shown in the top right corner. Below, alignments of the similar interfaces reported by Keren-Kaplan et al. are shown using only chain A of the query interface (matching chains are depicted in blue) and using both interface chains (green font). The other matches show alignments for high-scoring hits additionally found with PiMine using chain A of the query interface (orange font).

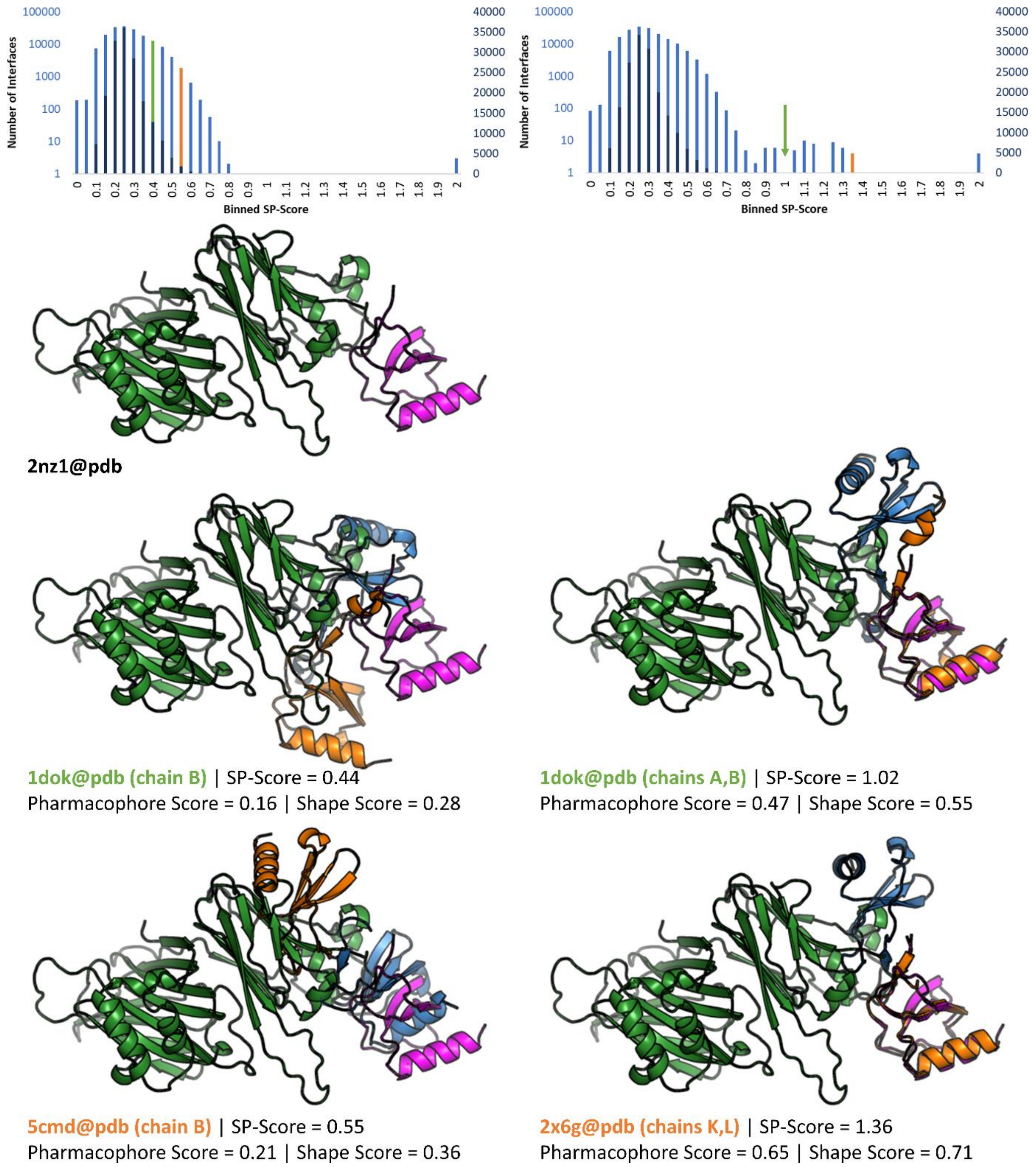

Figure S13. Matches found with a PiMine search for chain A of PDB entry 2nz1 (M3 protein of murid herpesvirus 4 in a complex with C-C motif chemokine 2) and interface chains A (green) and D (magenta). On the top, the score distributions for the *RunTimeSet* with the added similar interfaces from the work of Cheng and colleagues[2] is shown for a single-chain interface (left) and the interfaces of both chains (right). Below, alignments of the similar interfaces reported by Cheng et al. are shown using only chain A of the query interface (left) and using both interface chains (right). The matches at the bottom show alignments for high-scoring hits additionally found with PiMine using chain A of the query interface (left) and using both chain interfaces (right). The scores for these alignments are highlighted in green (reported example) and orange (high-scoring hit) in the distribution plots above.

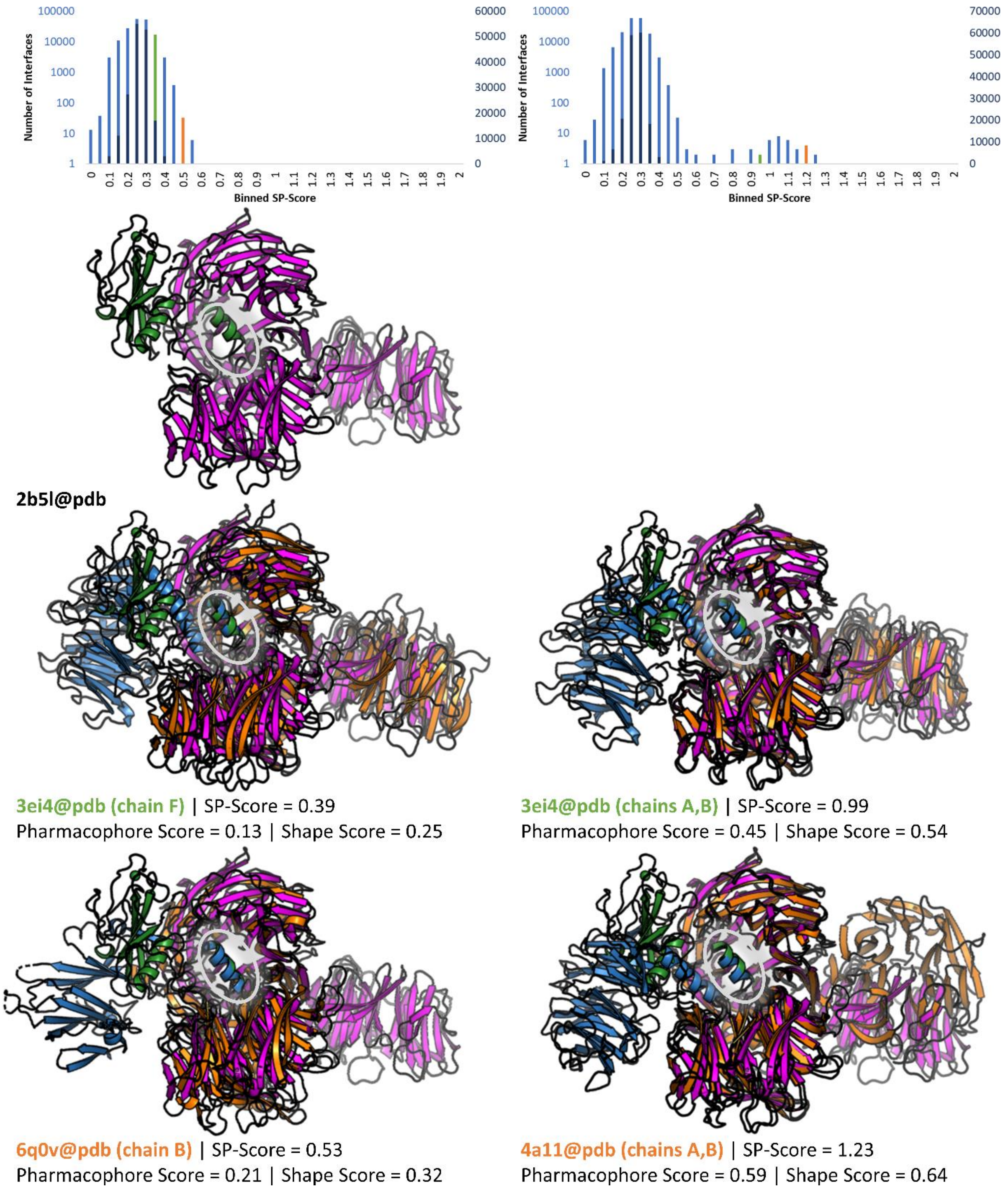

Figure S14. Matches found with a PiMine search for chain C of PDB entry 2b5l (Simian virus 5 nonstructural protein V in a complex with damage-specific DNA-binding protein 1) and interface chains C (green) and A (magenta). On the top, the score distributions for the *RunTimeSet* with the added similar interfaces from the work of Cheng and colleagues[2] is shown for a single-chain interface (left) and the interfaces of both chains (right). Below, alignments of the similar interfaces reported by Cheng et al. are shown using only chain C of the query interface (left) and using both interface chains (right). The matches at the bottom show alignments for high-scoring hits additionally found with PiMine using chain C of the query interface (left) and using both chain interfaces (right). The common helical motif discussed in the main paper is highlighted by a grey circle. The scores for these alignments are highlighted in green (reported example) and orange (high-scoring hit) in the distribution plots above.

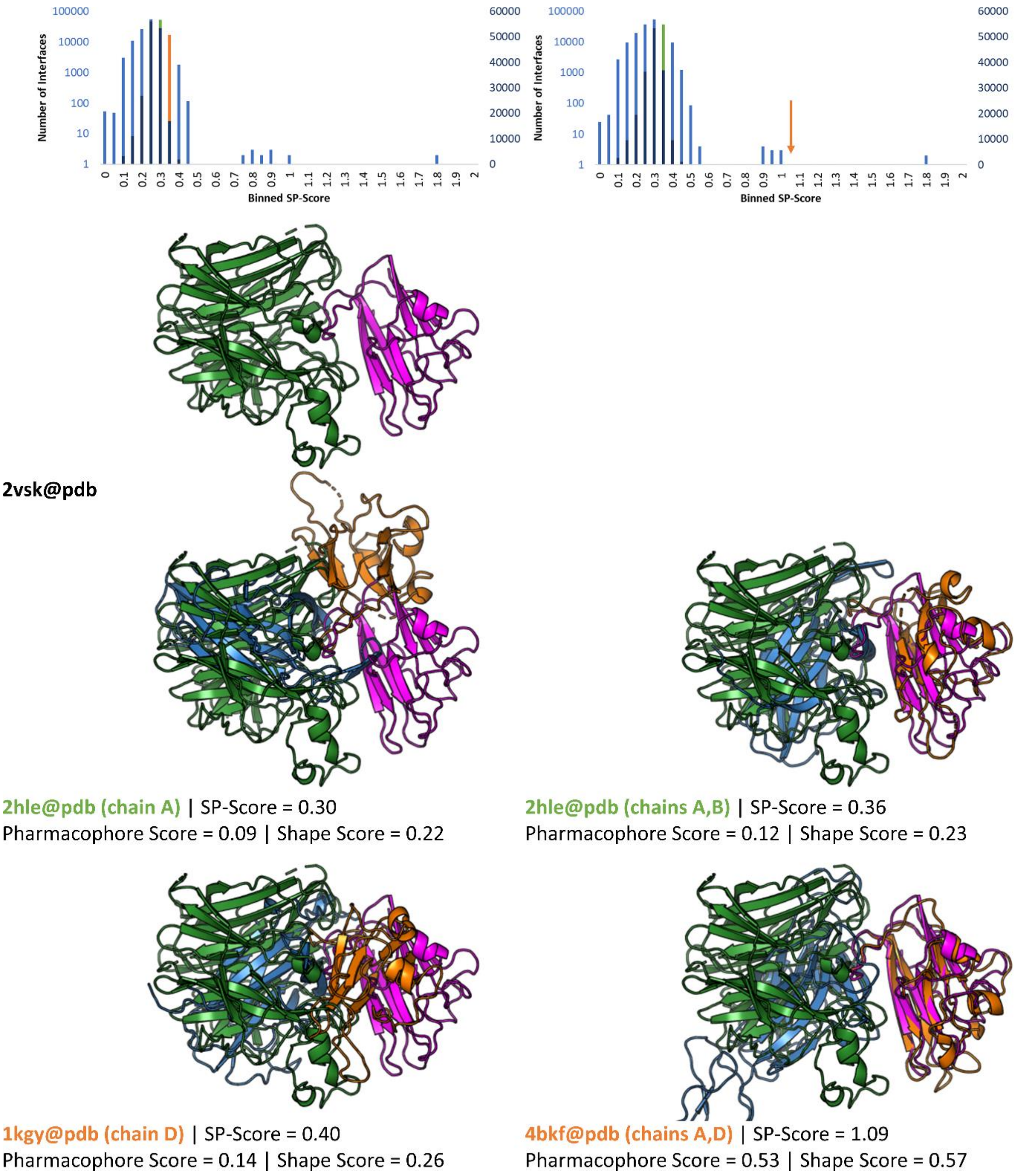

Figure S15. Matches found with a PiMine search for chain A of PDB entry 2vsk (Henda virus attachment protein glycoprotein G in a complex with ephrin-B2) and interface chains A (green) and B (magenta). On the top, the score distributions for the *RunTimeSet* with the added similar interfaces from the work of Cheng and colleagues[2] is shown for a single-chain interface (left) and the interfaces of both chains (right). Below, alignments of the similar interfaces reported by Cheng et al. are shown using only chain A of the query interface (left) and using both interface chains (right). The matches at the bottom show alignments for high-scoring hits additionally found with PiMine using chain A of the query interface (left) and using both chain interfaces (right). The scores for these alignments are highlighted in green (reported example) and orange (high-scoring hit) in the distribution plots above.

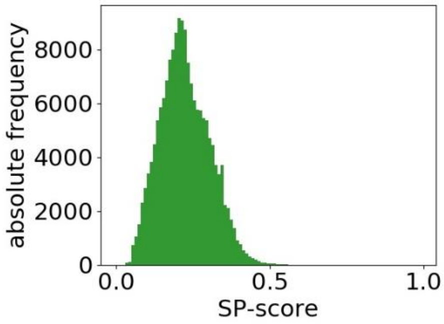

Figure S16. Score distribution for the PiMine search for similar interfaces to a predicted one of PDB entry 6cvz (human E3 ubiquitin-protein ligase RFWD3) in the *RunTimeSet*. The SP-scores were binned in 0.01 bins.

Table S1. PostgreSQL 14.6 custom parameters for PiMine runs on the SSD (on the *RunTimeSet*).

max_connections	20
shared_buffers	2 GB
effective_cache_size	8 GB
maintenance_work_mem	2 GB
checkpoint_completion_target	0.9
wal_buffers	16 MB
default_statistics_target	100
random_page_cost	1.1
effective_io_concurrency	200
work_mem	29127 kB
min_wal_size	100 MB
max_wal_size	2 GB
max_worker_processes	6
max_parallel_workers_per_gather	3
max_parallel_workers	6
max_parallel_maintenance_workers	3

Table S2. PostgreSQL 14.6 custom parameters for PiMine runs on the HDD (on the *RunTimeSet*).

max_connections	20
shared_buffers	2 GB
effective_cache_size	8 GB
maintenance_work_mem	2 GB
checkpoint_completion_target	0.9
wal_buffers	16 MB
default_statistics_target	100
random_page_cost	4
effective_io_concurrency	2
work_mem	29127 kB
min_wal_size	100 MB
max_wal_size	2 GB
max_worker_processes	6
max_parallel_workers_per_gather	3
max_parallel_workers	6
max_parallel_maintenance_workers	3

Table S3. Normalized enrichment factors of the methods iAlign, I2I-SiteEngine, and PiMine (scoring using both interfaces of the PPIs) on the *Dimer597* set.

Name	0.1%	0.5%	1%	2%	5%	10%	20%
iAlign-TM	0.97	0.98	1.0	1.0	1.0	1.0	1.0
iAlign-IS	0.95	0.98	0.99	1.0	1.0	1.0	1.0
I2I-SiteEngine	0.86	0.77	0.83	0.89	0.93	0.94	0.95
PiMine-SP (runtime-optimized)	0.86	0.60	0.66	0.71	0.77	0.80	0.84
PiMine-pharmacophore (runtime-optimized)	0.91	0.68	0.74	0.77	0.83	0.86	0.92
PiMine-shape (runtime-optimized)	0.78	0.52	0.56	0.61	0.68	0.74	0.79
PiMine-SP (accuracy-optimized)	0.88	0.62	0.68	0.73	0.78	0.81	0.86
PiMine-pharmacophore (accuracy-optimized)	0.88	0.68	0.72	0.75	0.82	0.88	0.91
PiMine-shape (accuracy-optimized)	0.80	0.55	0.60	0.64	0.71	0.76	0.81

Paragraph S1. PiMine Interface Input

Interfaces can be either loaded from the database by giving the PDB code and the identifiers of two interacting chains, or by giving a complex structure in the PDB or mmCIF file formats. In the case of the latter, either the PPI chain identifiers or a file formatted as PDB or mmCIF containing only the interface atoms must be provided. Complex structures given by the user are automatically pre-processed in the same way as in the database creation step.

Paragraph S2. External Benchmark Data Sets for Protein-Protein Interface Comparisons.

As an established protein-protein interface similarity data set, we used the so-called *Dimer597* set (https://sites.gatech.edu/cssb/ialign/) comprising 597 interfaces with 373 related pairs and 176,875 unrelated pairs and generated for the benchmarking of iAlign.[3]

The data set was created by first selecting dimers with chains of at least 200 residues and SCOP assignments, as the latter enable selecting biologically related protein domains.[4] Two protein-protein interfaces are considered as related if they share the same SCOP superfamily assignment and their overlap ratio is at least 30% based on the best-scored TM-align chain superposition, i.e., at least 30% of the total interface contacts have to overlap when comparing both interfaces. An overlapping contact is found if the distances between both contacts are below $d = 1.5[\min(|residues_{target}|, |residues_{query}|)]^{0.3} + 3.5$ and < 8 Å.[1] Interfaces are regarded as unrelated if the chain pairs of the aligned protein-protein complexes have differing SCOP superfamily assignments, a contact overlap ration of zero, and if less than 15% of the interface residues of the smaller interface in terms of number of residues are aligned by TM-align.

To verify whether PiMine recognizes interface similarities across unrelated protein dimer structures, we used a data set developed by Keskin et al.[5] All available PDB structures (July 18th, 2002) were filtered for multimers. To this end, two residues are regarded as interacting if the distance between any two atoms between residues from two chains is less than the sum of their van der Waals radii plus 0.5 Å. The authors removed all interfaces with less than ten interacting residues to exclude artificial crystal interfaces. Next, interfaces were extracted for the identified chain pairs by checking the distance between each Cα atom of the interacting residues and the surrounding Cα atoms. If this distance was less than 6 Å, the corresponding residue was assigned to the interface. Next, these approx. 20,000 interfaces are compared with each other using geometric hashing. The matching pairs are clustered by a heuristic iterative procedure. In each iteration cycle, the similarity definition is gradually relaxed. Of all resulting clusters, the sequences were compared using CLUSTAL W[6] and the BLOSSUM90 substitution matrix[7] within their cluster. To eliminate redundancy, interfaces whose sequence similarity is higher as 50% to at least one other interface of the current cluster are removed. Clusters with less than five members were also removed (https://web.archive.org/web/20200220060703/http://home.ku.edu.tr/~okeskin/INTERFACE/nonred-interface.list2). We generated all pairs of interfaces from the data set interfaces. Pairs annotated with the same cluster number were used as similar pairs. Otherwise, the pairs were classified as dissimilar. The resulting data set, which we call *Keskin* set, contains 103 clusters with 4,876 related interface pairs and 176,627 unrelated interface pairs.

References

(1) Keren-Kaplan, T.; Attali, I.; Estrin, M.; Kuo, L. S.; Farkash, E.; Jerabek-Willemsen, M.; Blutraich, N.; Artzi, S.; Peri, A.; Freed, E. O.; Wolfson, H. J.; Prag, G. Structure-based in silico identification of ubiquitin-binding domains provides insights into the ALIX-V:ubiquitin complex and retrovirus budding. EMBO J. **2013**, *32*, 538–551.

(2) Cheng, S.; Zhang, Y.; Brooks, C. L. PCalign: a method to quantify physicochemical similarity of protein-protein interfaces. BMC Bioinf. **2015**, *16*, 33

(3) Gao, M.; Skolnick, J. iAlign: a method for the structural comparison of protein–protein interfaces. Bioinformatics **2010**, *26*, 2259-2265.

(4) Murzin, A.; Brenner, S.; Hubbard, T.; Chothia, C. SCOP: a structural classification of proteins database for the investigation of sequences and structures. J. Mol. Biol. **1995**, *247*, 536-540.

(5) Keskin, O.; Tsai, C.-J.; Wolfson, H.; Nussinov, R. A new, structurally nonredundant, diverse data set of protein–protein interfaces and its implications. Protein Sci. **2004**, *13*, 1043-1055.

(6) Thompson, J. D.; Higgins, D. G.; Gibson, T. J. CLUSTAL W: improving the sensitivity of progressive multiple sequence alignment through sequence weighting, position-specific gap penalties and weight matrix choice. Nucleic Acids Res. **1994**, *22*, 4673-4680.

(7) Henikoff, S.; Henikoff, J. G. Amino acid substitution matrices from protein blocks. Proc. Natl. Acad. Sci. U. S. A. **1992**, *89*, 10915-10919.
